# Self‐management interventions for reducing challenging behaviors among school‐age students: A systematic review

**DOI:** 10.1002/cl2.1223

**Published:** 2022-03-07

**Authors:** Tyler E. Smith, Aaron M. Thompson, Brandy R. Maynard

**Affiliations:** ^1^ Department of Educational, School, & Counseling Psychology, Missouri Prevention Science Institute University of Missouri Columbia Missouri USA; ^2^ School of Social Work, Missouri Prevention Science Institute University of Missouri Columbia Missouri USA; ^3^ School of Social Work Saint LouisUniversity St. Louis Missouri USA

## Abstract

**Background:**

Challenging classroom behaviors can interfere with student social and academic functioning and may be harmful to everyone in schools. Self‐management interventions within schools can address these concerns by helping students develop necessary social, emotional, and behavioral skills. Thus, the current systematic review synthesized and analyzed school‐based self‐management interventions used to address challenging classroom behaviors.

**Objectives:**

The current study aimed to inform practice and policy by (a) evaluating the effectiveness of self‐management interventions at improving classroom behaviors and academic outcomes and (b) examining the state of research for self‐management interventions based on existing literature.

**Search Methods:**

Comprehensive search procedures included electronically searching online databases (e.g., EBSCO Academic Search Premier, MEDLINE, ERIC, PsycINFO), hand‐searching 19 relevant journals (e.g., *School Mental Health*, *Journal of School Psychology*), reference‐list searching 21 relevant reviews, and searching gray literature (e.g., contacting authors, searching online dissertation/theses databases and national government clearinghouses/websites). Searches were completed through December of 2020.

**Selection Criteria:**

Included studies employed either a multiple group‐design (i.e., experimental or quasi‐experimental) or single‐case experimental research design and met the following criteria: (a) utilized a self‐management intervention, (b) conducted in a school setting, (c) included school‐aged students, and (d) assessed classroom behaviors.

**Data Collection and Analysis:**

Standard data collection procedures expected by the Campbell Collaboration were used in the current study. Analyses for single‐case design studies incorporated three‐level hierarchical models to synthesize main effects, and meta‐regression for moderation. Further, robust variance estimation was applied to both single‐case design and group‐design studies to account for dependency issues.

**Main Results:**

Our final single‐case design sample included 75 studies, 236 participants, and 456 effects (i.e., 351 behavioral outcomes and 105 academic outcomes). Our final group‐design sample included 4 studies, 422 participants, and 11 total behavioral effects. Most studies occurred in the United States, in urban communities, in public schools, and in elementary settings. Single‐case design results indicated that self‐management interventions significantly and positively impacted both student classroom behaviors (LRRi = 0.69, 95% confidence interval [CI] [0.59, 0.78]) and academic outcomes (LRRi = 0.58, 95% CI [0.41, 0.76]). Single‐case results were found to be moderated by student race and special education status, whereas intervention effects were more pronounced for African American students (*F* = 5.56, *p* = 0.02) and students receiving special education services (*F* = 6.87, *p* = 0.01). Single‐case results were not found to be moderated by intervention characteristics (i.e., intervention duration, fidelity assessment, fidelity method, or training). Despite positive findings for single‐case design studies, risk of bias assessment indicated methodological shortcomings that should be considered when interpreting findings. A significant main effect of self‐management interventions for improving classroom behaviors was also revealed for group‐design studies (*g* = 0.63, 95% CI [0.08, 1.17]). However, these results should be interpreted with caution given the small number of included group‐design studies.

**Implications for Policy, Practice, and Research:**

The current study, conducted using comprehensive search/screening procedures and advanced meta‐analytic techniques, adds to the large amount of evidence indicating that self‐management interventions can be successfully used to address student behaviors and academic outcomes. In particular, the use specific self‐management elements (i.e., self‐determining a performance goal, self‐observing and recording progress, reflecting on a target behavior, and administering primary reinforcers) should be considered within current interventions as well as in the development of future interventions. Future research should aim to assess the implementation and effects of self‐management at the group or classroom‐level within randomized controlled trials.

## 1 PLAIN LANGUAGE SUMMARY

### 1.1 School‐based self‐management interventions improve behavioral and academic outcomes for K‐12 students with challenging behaviors

School‐based self‐management interventions targeting students with challenging behaviors on average have positive effects across behavioral (i.e., prosocial, on‐task, disruptive, following directions) and academic outcomes (i.e., achievement, work completion). Results were found to be most impactful for African American students, and students receiving special education services.

### 1.2 What is this review about?

Approximately 20% of students repeatedly display challenging classroom behaviors (e.g., off‐task, disruptive behavior). Students exhibiting challenging classroom behaviors have difficulties achieving academic success and may indirectly harm the learning of classroom peers.

This review provides support for the use of school‐based self‐management interventions—including self‐assessment, self‐monitoring, and self‐evaluation practices—for children with challenging behaviors.

Self‐management interventions targeted a range of classroom behaviors (i.e., prosocial behaviors, on‐task behaviors, disruptive behaviors, and following directions).

This review provides support for the use of school‐based self‐management interventions—including self‐assessment, self‐monitoring, and self‐evaluation practices—for children with challenging behaviors. Self‐management interventions targeted a range of classroom behaviors (i.e., prosocial behaviors, on‐task behaviors, disruptive behaviors, and following directions).

This Campbell systematic review examines the effects of self‐management interventions to address student behaviors and academic outcomes in schools. The review summarized and analyzed evidence from 75 single‐case design studies and four group‐design studies, of which three were experimental and one was quasi‐experimental.

### 1.3 What studies are included?

Included studies examined self‐management interventions for students with challenging classroom behaviors. For inclusion, studies had to identify the use of a self‐management intervention, be conducted in a school setting, include school‐aged students, assess challenging behavior outcomes, and include one of the following research designs:

(1) Group‐design experimental or quasi‐experimental studies (*n* = 4).
(2) Single‐case design studies (*n* = 75).

### 1.4 What are the main findings of this review?

Self‐management interventions significantly and positively impact student classroom behaviors as indicated by moderate effects revealed for both single‐case and group‐design studies.

Results of single‐case design studies additionally indicated that self‐management interventions significantly and positively impacted all challenging behaviors assessed (i.e., on‐task behavior, prosocial behaviors, disruptive behaviors, and following directions) and academic outcomes (i.e., achievement and work completion).

Single‐case effects were also found to be more meaningful for African American students in comparison to other races, and for students receiving special education services in comparison to students in regular classrooms.

### 1.5 What do the findings of this review mean?

This review provides support for self‐management interventions as a means to successfully address student challenging classroom behaviors. Additionally, self‐management interventions significantly improve children's academic achievement and work completion.

These conclusions are primarily based on single‐case design studies, as the small number of included group‐design studies makes it difficult to make accurate determinations.

That said, some methodological shortcomings of included single‐case design studies indicate that presented findings should be read with caution. Additionally, many single‐case design studies were not included in the current review due to not meeting minimum design/quality guidelines. More high‐quality research is needed, especially utilizing experimental group‐designs, to make further and more valid conclusions.

### 1.6 How up‐to‐date is this review?

The review authors searched for studies published up to December of 2020.

## 2 BACKGROUND

### 2.1 Description of the problem or condition

Most parents and teachers agree that students need to exhibit appropriate social behaviors to achieve academic goals; however, approximately 20% of students, or 3‐4 students in the average classroom, repeatedly display challenging behaviors that interfere with normal academic and social development (Brauner & Stephens, 2006; Bushaw & Lopez, 2010; Satcher, 2004; Walker et al., 2004). Challenging behaviors at school can manifest under many conditions and in various locations within a school (Flower et al., 2014). Challenging student behaviors can include a range of acts that may (a) interfere with social and academic functioning and (b) harm a child, his or her peers, or adults within the school. Researchers have identified a number of challenging behaviors at school including defiance, disrespect, harassment, verbal and physical aggression (Kaufman et al., 2010), violating classroom rules, talking without permission, getting out of one's seat (Walter, Gouze, & Lim, 2006), and general distractibility and issues following directions (Harrison et al., 2012). Because research often distinguishes between subtypes of challenging behaviors, we specify three broad subtypes: (a) direct and indirect forms of aggression (e.g., hitting, name calling, spreading rumors; Dodge & Coie, 1987; Leff & Crick, 2010; Parke & Slaby, 1983); (b) overt and covert antisocial behaviors (e.g., stealing, bullying, lying, cheating); and (c) low intensity acts of insubordination (e.g., noncompliance, withdrawal, refusal to cooperate, impulsivity, inattention, off‐task; Kaiser & Rasminsky, 2009).

Challenging student behaviors are harmful to everyone in schools—including students who exhibit the behaviors and their peers and teachers. Students who exhibit challenging behaviors are frequently removed from class, which interrupts instruction, exacerbates academic difficulties, and increases the likelihood of school failure and dropout (Gresham et al., 2000; Nelson et al., 2004). Peers of disruptive students are adversely affected by the behaviors due to lost instructional opportunities (U.S. Department of Education [USDOE], 2006). Observational studies indicate challenging behaviors contribute to a loss of four hours of instruction per week in the average classroom or about 144 h per student over the academic year (Walker et al., 2004). Lastly, teachers experience increased stress and burnout associated with managing challenging behaviors (Brouwers & Tomic, 2000; Clunies‐Ross et al., 2008; Grayson & Alvarez, 2008; Hastings & Bham, 2003; Joseph & Strain, 2003). A survey of highly‐qualified teachers suggested that 53% of those who requested transfers and 44% of those who quit teaching cited challenging student behaviors as their primary reason for the decision (USDOE, 2005). Because challenging behaviors adversely impact everyone in schools, it is vital that school professionals assist students with challenging behaviors to learn adaptive social, emotional, and behavioral skills.

Research suggests school‐based programs that promote competencies in social, emotional, and behavioral skills hinge on the development of five interrelated concepts: social‐awareness, self‐awareness, self‐management, relationship skills, and problem solving (Bridgeland et al., 2013). Also called social, emotional, noncognitive, or soft skills, exposure to these skills increases the likelihood that students with challenging behaviors will experience better proximal school‐related and distal life‐course outcomes (Durlak et al., 2011; Heckman & Kautz, 2012; Wilson & Lipsey, 2007). For example, programs and practices promoting the development of the aforesaid skills are related to improvements in social functioning (ES = 0.69), attitudes toward school (ES = 0.24), behavioral problems (ES = 0.26), emotional stability (ES = 0.28), and academic performance (ES = 0.28; Durlak et al., 2011).

To facilitate the development of social, emotional, and behavioral skills, researchers and educators increasingly recognize the importance of autonomy support as an intervention mechanism (Algozzine et al., 2001; Field et al., 1998; Lane et al., 2010). Autonomy refers to a sense of self‐management (Deci & Cascio, 1972; Deci & Ryan, 2011; Deci et al., 1975; Wigfield et al., 2007, 2008). Authority figures who endorse and enable the development of skills and opportunities required for self‐management engage in autonomy support strategies (Deci & Ryan, 2011; Field et al., 1998). Strategies that integrate principles of autonomy support include—but are not limited to—instruction in decision making, problem solving, goal setting, self‐awareness, self‐assessment, self‐evaluation, self‐management, and self‐monitoring (Algozzine et al., 2001; Lane et al., 2010; Wehmeyer & Schwartz, 1997). Autonomy support strategies also facilitate improved student–teacher relations (Wentzel et al., 2007). Improved student–teacher relations diminish challenging behaviors and makes disciplining students who display those behaviors more effective (Hamre & Pianta, 2003).

In summary, promoting the development of competencies in social, emotional, and behavioral skills requires on‐going and quality instruction in self‐awareness, social awareness, self‐management, relationship skills, and problem‐solving skills. Educators can nurture and cultivate the development of these valuable skills through autonomy support strategies that propagate an increased sense of self‐management in students. Though many strategies impart social emotional skills and promote student autonomy separately, a self‐management intervention combines social and emotional skills with autonomy support in a single approach.

### 2.2 Description of the intervention

The review focuses on the effectiveness of school‐based *self‐management* (SM) interventions—a widely‐used intervention to address disruptive and challenging behaviors in school settings. The principles of SM were initially developed from the field of behavioral psychology. SM interventions are strongly rooted in behavior analytic methods, and later, have been influenced by cognitive‐behavioral theories (Mahoney, 1970). Though SM interventions are referred by many names (e.g., self‐control, effortful control, self‐regulation), SM is defined as a set of strategies that students are trained in to assess, monitor, and/or evaluate their own behavioral performance (Briesch & Chafouleas, 2009; Cole et al., 2000; Fantuzzo et al., 1988; Maggin et al., 2013; Rothbart & Rueda, 2005; Shapiro & Cole, 1994; Shapiro et al., 2002). More specifically, Fantuzzo and colleagues (1988) suggest a SM intervention includes one or a combination the following elements:

1. self‐selecting a target behavior
2. self‐defining the target behavior
3. self‐determining a performance goal
4. self‐identifying reinforcers
5. self‐prompting a reflection of behavior
6. self‐observing a target behavior
7. self‐recording the observations
8. self‐charting the observations
9. self‐appraising performance
10. self‐administering primary reinforcers
11. self‐administering secondary reinforcers

#### 2.2.1 The SM procedures

The SM procedures consist of students engaging in one or a combination of the 11 processes listed above that constitute a SM intervention. Although procedural aspects would certainly be taught during the training stage, the SM procedural stage directly refers to the cognitive and behavioral processes a student would be expected to engage in during the actual implementation of a SM intervention. The procedural stage may include any one or a combination of the 11 SM elements listed above (Hallahan & Sapona, 1983; Rutherford Jr, Quinn, & Mathur, 2004; Vaughn et al., 2011).

During the self‐assessment phase, students may *self‐select*, *self‐define*, and *self‐determine* reasonable performance goals to address a target behavior. Even if students are only self‐monitoring on‐ or off‐task behavior, they must first select and define a behavior. Ideally, the behavior will be one that occurs at a frequency or rate that allows it to be observed or detected at regular intervals. That is, if a behavior is low frequency then it is unlikely to register or be observed to a degree that self‐monitoring will capture useful information about the behavior. Once a student has identified and defined a problem behavior, a goal may also be set to reduce the problem or increase the performance of a preferred replacement behavior. Using observable and measurable terms (i.e., frequency, duration, and/or severity of the behavior), a student may operationally define a goal using positive language (i.e., I will increase my work completion) or negative language (i.e., I will not tap my pencil on my desk). Though some researchers argue that to be considered a true SM intervention, students should directly participate in each of the 11 SM steps, many studies report a process whereby educators select, define, and set performance goals for students (Briesch & Chafouleas, 2009). Students or teachers may also identify reinforcers at this stage should the student achieve the goal. Once the student has selected, defined, and set a goal to address a behavior, the student is ready to self‐monitor his or her performance.

During the self‐monitoring phase, students first *self‐prompt* or are externally prompted to self‐observe. During the *self‐observation*, a student reflects upon his or her performance and discriminates whether he or she displayed the target behavior during the interval. During the *self‐recording* process, a student would physically record the observation on a schedule (see Tables 1 and 2) to indicate the presence or absence of the target behavior. Following the self‐monitoring phase, a student then may evaluate his or her own progress.

**Table 1:** Record example for a young student

	Before lunch	After lunch
Raise hand	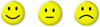	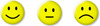
Stayed in seat	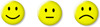	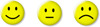

**Table 2:** Record example for an older student

Time	Completed work		Accepted direction		Stayed in assigned area	
8:00–8:30	Yes	No	Yes	No	Yes	No
8:31–9:00	Yes	No	Yes	No	Yes	No
9:01–9:30	Yes	No	Yes	No	Yes	No
9:31–10:00	Yes	No	Yes	No	Yes	No
10:01–10:30	Yes	No	Yes	No	Yes	No

During the self‐evaluation of performance, students self‐chart their performance by calculating percentages or creating graphic images of the data collected (DiGangi et al., 1991). Using the percentages, graphs, or charts, the student can *self‐appraise* or compare his or her results to a predefined goal, prior observational data, using teacher or other third‐party observations, or combination of those standards (Thompson & Webber, 2010). Using the standards, students can determine whether their performance met the standard and—if relevant—*self‐administer* selected reinforcers. The performance comparisons may also be used to develop new performance goals before the process is then iteratively repeated.

#### 2.2.2 The SM student training

Considerations for training students in SM are provided by Cole et al. (2000), Shapiro et al. (2002), Strayhorn (2002b), and Lane et al. (2010)—however very little empirical research identifies which of these considerations are more important than others.

Some research suggests training is more effective when it (a) is sequenced, (b) uses active learning modalities such as modeling and rehearsal, (c) is focused on individual needs, and (d) explicitly defines the behavioral skills required to engage in SM (Durlak et al., 2011). Training is also enhanced when students have opportunities to practice the procedures of SM (Schunk & Zimmerman, 1998; Strayhorn, 2002a, 2002b), and when those practice sessions result in specific and formative feedback aimed at improving accuracy (Shute, 2008). Some research also indicates that feedback should also be supportive when affirming or correcting student SM efforts (Bandura, 1994; Dweck, 1975; Mueller & Dweck, 1998; Zimmerman, 1989). Praise for effort communicates that failure is a part of learning and effort matters more than achievement, effectively lowering the effect of appraisal on performance of a required task (Dweck, 2006). Such messages also engender the development of quality relations between students and teachers (Wentzel, 1991) and encourage children to practice and integrate SM skills (Lewis, 2000).

There are three possibilities when determining the focus of training for a SM intervention. The first possibility is to determine whether the student can perform a task. If the student cannot perform the task or achieve the desired outcome, then he or she will require direct instruction in the basic steps to perform that task. If the student has the ability to perform a task but requires assistance with doing the task fluently, smoothly, or with more confidence, a second possibility can be considered. In this instance it must be determined whether the student requires additional instructional supports, more practice, or both. Lastly, if the student has the capacity to perform the task fluently but refuses or is not motivated, then the student may need support or reinforcement to engage in the task. Either way, studies suggest that students may be trained in SM and that training can mitigate any of the three performance barriers listed here (Cole et al., 2000; Lane et al., 2010; Shapiro et al., 2002).

Given the number of cognitive and behavioral skills required to engage in SM, a number of considerations raised by Cole and colleagues (2000) serve as a helpful guide to illustrate how student training in SM will likely vary across studies:

1. What type of students will SM be used with?
2. What type of outcomes will SM be used with?
3. What type of setting will SM be used in?
4. What type of prompt is suitable for the setting?
5. What type of recording device is suitable?
6. What type of reinforcement should be used?

#### 2.2.3 Type of students

A slew of studies have suggested that SM interventions are feasible and effective at improving behavioral outcomes for males and females, students from a variety of racial and ethnic groups, and students in elementary through high school grades (Briesch & Chafouleas, 2009; Shapiro et al., 2002). Studies have suggested SM interventions are effective for both students without disabilities (Wood et al., 1998) and for those with a range of disabilities—including autism (Koegel et al., 1992), developmental delays (O'Connell et al., 2009), attention‐deficit/hyperactivity disorder (ADHD) learning disabilities (Shimabukuro et al., 1999), emotional and behavioral disorders (EBD; Thompson & Webber, 2010; Thompson, 2012), and mild or moderate intellectual disability (Boswell et al., 2013; Hughes et al., 2002; Smith et al., 1992). That said, individuals with severe or profound intellectual disability that have significant cognitive impairments, difficulties with implementing tasks independently, and limited or no verbal behavior, may not benefit from SM (Ganz & Sigafoos, 2005; Kahn, 1996). Given that SM can involve, multiple, sequenced, independent tasks and metacognitive strategies, it may not be a realistic or economic approach for individuals with severe or profound intellectual disability (Lancioni & O'Reilly, 2001; Shapiro, 1981).

#### 2.2.4 Type of outcomes

The research underlying the effectiveness of SM suggests the intervention is effective at improving both academic and behavioral outcomes (Briesch & Chafouleas, 2009; Mooney et al., 2005). Regarding academic outcomes, SM has been shown to improve academic performance and rates of work completion and accuracy (Carr & Punzo, 1993; Miller et al., 1989; Mooney et al., 2005). With regard to behavioral outcomes, studies suggest SM may be used to improve attention and compliance (e.g., following directions), and reduce impulsivity and externalizing behaviors (e.g., talking out, out of seat). Studies also suggest SM has been used to decrease more common acts of insubordination such as off‐task behaviors (Blick & Test, 1987; Dunlap & Fox, 1999; Hallahan & Sapona, 1983; Prater et al., 1991; Webber et al., 1993) as well as acts associated with aggressive and antisocial behaviors (Bennett & Gibbons, 2000; Todd et al., 1999).

#### 2.2.5 Type of setting

A variety of settings in a school may condition a SM intervention. Since students spend the majority of their time in the classroom, a majority of studies examine the effects of SM on classroom behaviors. However, some studies have also examined the effects of SM on behavior on the playground (Koegel et al., 1992), in gym class (Zimmerman & Kitsantas, 1996), and in the hallways or during other unstructured transitions (Connell et al., 1993). The type of setting is often a result of the target behavior and contextual factors associated with the behavior. Because the setting or context will vary, studies will vary in the types of behavior and prompts used.

#### 2.2.6 Type of prompt

Many different prompts are used in the self‐monitoring phase of a SM intervention. Prompts, broadly speaking, come in two forms: internal and external. Internal, also referred to as a self‐prompt, generally requires a student to remind herself to reflect on her own behavior. However, the reliability of internal prompts is not well‐established and is questionable given the challenges faced by children with attentional and behavioral issues. As such, many studies rely on external prompts (Cole et al., 2000; Shapiro et al., 2002).

External prompts may take the form of a verbal or nonverbal cue delivered by an adult (e.g., verbal reminder, hand signal) or an electronic device (e.g., a watch, a timer). Some studies have used tape recorders and headphones to prompt students (DiGangi, Maag, & Rutherford Jr, 1991; McDougall & Brady, 1998). More recently, studies are beginning to examine the use of personal digital devices as prompts (i.e., laptops, tablets, personal digital assistants, mobile phones, and digital vibrating devices). One possible benefit offered by personal digital devices is that they address issues of reliability during the self‐monitoring phase of a SM intervention. Findings suggest the prompts delivered by these devices do not disrupt other students (e.g., vibrating devices), allow practitioners to vary the self‐monitoring schedule to fit an individual student's need, and improve the accuracy of self‐observation and self‐recording (Amato‐Zech et al., 2006). The use of mobile technology in the research is beginning to explore the use of digital devices to integrate external prompts with real time digital data collection of SM observations (Fjeldsoe et al., 2009; Gulchak, 2008; Mitchem et al., 2007). Obviously, the type of prompt has implications for reliability of the monitoring element of a SM intervention—although some research suggests SM is effective regardless of the accuracy and reliability of the self‐observations (Cole et al., 2000). Regardless, the type of recording device will vary across the studies included in the review and will impact how students are trained in SM.

#### 2.2.7 Type of recording device

The type of recording device refers to the characteristics of the tool used to record SM observations (Cole et al., 2000). Broadly speaking, two characteristics are used to describe a monitoring device—interval frequency and observational response options. To increase the accuracy of monitoring data—and arguably the effectiveness of a SM intervention—the recording device should be simple. The characteristics of the device are likely to vary along several lines as determined by student needs and behaviors.

First, a device should be simple, available, portable, and have utility in multiple school settings (e.g., hallways, classroom, playground, gym class; Cole et al., 2000). Availability and portability increase the device's utility. However, the target behavior and how the behavior is operationally defined will also condition the utility of the device across multiple school settings. That is, some behaviors are just not relevant to all settings or occur in the presence of certain other factors. Lastly, the cost impacts the choice of device in practice and research. Generally, paper‐and‐pencil interval recording devices are used in most SM studies because they are easily manipulated, highly accessible, and are low‐cost (Lane et al., 2010; Shapiro et al., 2002).

Because studies examine the effects of SM with the full range of school‐aged students as well as across students with varying disability types, the age and ability of a child are important considerations for determining the format of a recording device. Some devices may only record the presence of an on‐ or off‐task behavior (Bolstad & Johnson, 1972; Harris et al., 2005; Harris, 1986; Shapiro et al., 2002). Other studies may have specific behavioral goals (Thompson & Webber, 2010). However, most studies use time interval formats with multiple intervals spaced at equal time points.

Tables 1 and 2 provide some selected examples of the broad number of interval SM recording devices. While there are many possible formats for the device, Table 1 exemplifies a device useful with young students or those with learning impairments (i.e., few intervals, responses with pictorial options). The example in Table 2 may be used with older students. In this example, there are multiple target behaviors and multiple recording intervals to address an array of complex and competing behaviors.

#### 2.2.8 What type of reinforcers should be used?

Using contingency reinforcers alongside a SM intervention may improve the success of the intervention. For example, requiring a student to meet his or her predetermined goal to earn a positive reinforcer (e.g., extra time at recess or playing a game with a peer) or a negative reinforcer (e.g., earn a pass on completing an assignment) has been shown to improve outcomes (Glynn et al., 1973; Webber et al., 1993). Studies also suggest when SM goals are achievable and the contingencies are provided immediately upon goal attainment, SM appears to be more effective (Lane et al., 2008). In summary, there are many aspects to student training and only a few of which are listed here. However, it is generally agreed that students should be trained in the skills needed for SM before they actually engage in following SM procedures.

In summary, the review will examine SM interventions, a widely‐used cognitive behavioral intervention that appears to be effective for academic and behavioral outcomes. Though implementing a SM intervention appears straightforward, there are many variations in practice surrounding training and implementation of a SM intervention. Regardless of these variations, we suggest a SM intervention is best defined as a set of strategies that train students to assess, monitor, and evaluate their own behavioral performance. As such, SM consists of two stages: a training stage and a procedural stage. Though some resources are available to suggest best practices and considerations for training students to engage in SM procedures, no manualized SM programs are available that sequence empirically supported elements of student training, which may improve outcomes. As such, it is expected that the type, quality, and degree of student training will vary greatly across studies of SM interventions. In addition, the SM procedural stage will also vary based upon many contextual and child‐specific features.

### 2.3 How the intervention might work

There are several important behavioral principals or mechanisms of change underlying a SM intervention. To begin, behavior change can occur by the very function of engaging in the self‐monitoring aspect of a SM intervention (Nelson & Hayes, 1981). Also known as the *reactivity* principle, the simple act of collecting SM data regarding one's own behavioral functioning is thought to alter the behavior itself. For example, as observed in studies of self‐regulated learning (Bandura, 2005; Cleary & Zimmerman, 2004), students who self‐monitored their performance on a number of math problems were systematically introduced to a heightened awareness of the number of problems answered correctly. Change was hypothesized to occur as a direct result of internal reward mechanisms that influence behavior change. That is, the simple act of observing and recording one's own performance informs and influences reward centers, which alters motivation and behavior (Shapiro et al., 2002). The reactivity phenomenon has also been observed in a variety of other research areas. For example, weight loss was observed in studies where participants monitored daily caloric intake and types of foods eaten without engaging in dieting interventions (Boutelle & Kirschenbaum, 2012; Butryn et al., 2012). The reactivity phenomenon has also been observed in studies of SM interventions with persons who have substance and alcohol abuse disorders (Bien et al., 2006; Simpson et al., 2005). Although it may appear, on the surface, that no discernible extrinsic reinforcers are present during the SM procedure—the very act of reflecting on behavior, collecting data on behavior, and using that data to evaluate performance over time is a metacognitive activity that alters the targeted behaviors.

Another mechanism related to the theory of change underlying SM—one closely related to the concept of reactivity, self‐management, self‐awareness, and intrinsic motivation—is the concept of *perceived autonomy* (Deci & Ryan, 2011; Deci et al., 1975; Wigfield, Eccles, Schiefele, Roeser, & Davis‐Kean, 2007). In a SM strategy, students engage in an act of “perceived autonomy.” That is, students are encouraged to self‐assess, self‐monitor behaviors, and self‐evaluate specific behaviors (Algozzine et al., 2001; Field et al., 1998; Wehmeyer & Schwartz, 1997). Because behavior change is often a “top down” activity that is prepared, planned, and applied to students by teachers, supporting student autonomy through the use of a SM intervention improves perceived ownership and motivation to engage in the intervention, which leads to an increased likelihood of positive outcomes (Lane et al., 2010). Indeed, a variety of studies have shown that when teachers engage in autonomy support strategies (e.g., choice making, goal setting, instruction in self‐observation, instruction in self‐control), participants perform tasks consistently better than tasks where autonomy is not supported (DeCharms, 1984). In short, SM is an autonomy support activity that provides students with choice and, as such, they experience increased levels of perceived autonomy.

Furthermore, because SM is an iterative process, students are provided with increased opportunities to practice skills. Opportunities to practice novel skills leads to an increased sense of *self‐efficacy* or *competency* surrounding the completion of a required behavioral task (Eccles et al., 1997; Niemiec & Ryan, 2009). With increased competencies, students are more likely to adopt and integrate those external requirements into their repertoire of internalized skills and values (Gagné, 2003). Furthermore, autonomy support, relevant instruction, and increased opportunities to practice and develop competencies have been shown to improve relationships between students and teachers. That is, autonomy support is an important mediator shown to facilitate healthy and trusting Student–teacher relationships (Connell et al., 1993; Cox & Williams, 2008; Hamre & Pianta, 2003; Wentzel, 1993, 2002; Wentzel et al., 2007). Quality relations between students with challenging behaviors and their teachers diminishes challenging behaviors (Wentzel et al., 2007) and makes disciplining students who do engage in challenging behaviors more effective (Hamre & Pianta, 2003).

### 2.4 Why it is important to do the review

At the time when we drafted the protocol for this review in 2013, there were five known reviews that examined the impact of SM on student behavioral or academic outcomes. At the time we drafted the report for this review, our procedures uncovered a total of 16 reviews of SM interventions—excluding the five previously known reviews. In total—there are presently 21 published reviews of SM interventions. Fourteen of these reviews involve quantitative synthesis, whereas seven present descriptive summaries of intervention outcomes, components, and other study characteristics. A majority of these reviews (19) examine the impact of SM on behavioral outcomes while two examine the impact of SM on academic outcomes. More specifically, 11 of these reviews focus on SM interventions for students with challenging behaviors; seven reviews focus on SM interventions for students with learning or behavioral/emotional disabilities or attention related diagnoses; and three reviews focus on the use of SM interventions with children who have autism spectrum disorders (ASD). The systematic reviews, taken together, strongly suggest that a SM intervention impacts desirable behavioral and academic outcomes. Since the development of the protocol for the present review in 2013—nine reviews have been published since 2014. Regardless, in examining these and other reviews uncovered during the search and completion of this study, other SM reviews demonstrate similar limitations that align with the original reason that prompted the current proposed review.

The most noteworthy limitation of SM reviews has to do with the methods used to generate effect sizes. Although prior reviews did not benefit from emerging methods to generate standardized summary effects, the methods used in the studies likely overestimate the effects of SM. Though a great deal of debate surrounds the best approach for synthesizing findings from single case designs (SCD), some of the prior reviews combined single subject and group studies in one review, combined multiple baseline and intervention phases, and used the “no assumptions” approach for estimating summary effects (Busse et al., 1995). The no assumptions effect size is estimated by subtracting the mean of the baseline from the intervention mean and dividing the result by the baseline standard deviation. Such summary estimates, when not properly accounted for, inflate effect size estimates, evidenced in part by summary effects of SM ranging anywhere from 4.19 to 30.25 (Briesch & Chafouleas, 2009; Fantuzzo & Polite, 1990; Mooney et al., 2005). Two reviews that included the same group of studies (i.e., Briesch & Chafouleas, 2009; Maggin et al., 2013) relied on two complimentary yet limited approaches to estimate summary effects. The first statistic used in both reviews was the percent of nonoverlapping data (PND)—a common metric developed for use in single subject studies (Scruggs et al., 1987). The drawback of PND is that the approach does not account for the autocorrelation inherent in single case studies. Autocorrelation occurs when behavior at one point in time is influenced by or highly correlated with behavior at another point in time. When this happens, results can lead to falsely showing a treatment effect that is not actually present. Furthermore, PND does not account for baseline trends that may explain improvements observed during the treatment phase. For instance, it is possible that a student's behavior may be improving during baseline (i.e., as indicated by a positive upward trend on a graph), and that this trend in improved behavior would continue regardless of if a student receives an intervention. The second statistic used in both reviews was a standard mean difference effect generated using ordinary least squares models with fixed effects. Such approaches do not account for the wide heterogeneity observed when (a) combining phases within single case studies, (b) combining effects across single case studies, or (c) combining single case and group‐designs in single summary effects. The Mooney et al. (2005) review used a standard mean difference to estimate summary effects. However, Mooney (2005) only included the average of the last three data points in each phase—a practice that has been shown to inflate summary effect sizes (Olive & Smith, 2005). Since this proposal was drafted in 2013, there have been several advancements related to effect size indices for SCD studies. Based on these emerging indices and the structure of our data, we believe the log response ratio developed by Pustejovsky (2018) is the best and most advanced option for the current study. To our knowledge, this is the first SM review to utilize this effect size index.

A second limitation of the prior reviews hinges on the search procedures used in each of the studies. That is, the prior studies relied upon (a) the same search terms and (b) the same two databases (i.e., PsycINFO and ERIC). In addition, prior reviews included no “gray” literature strategies to include effects of published and unpublished sources not commercially controlled. By extending our search procedures via searching multiple online databases (i.e., Academic Search Premier, Dissertation Abstracts International, ERIC, MEDLINE, PsycINFO, Social Service Abstracts, Social Work Abstracts, and Sociological Abstracts), hand searching 19 journals, searching gray literature, and reference‐list searching previous SM reviews, we believe our combined approaches to be the most comprehensive to date. In particular, our search procedures located nearly 10,000 records that were searched for potential inclusion. By comparison, Fantuzzo and Polite (1990) initially located 987 results, Briesch and Chafouleas (2009) located 794 results, and Maag (2019) located 416 results. Although most records were excluded, we feel that our search strategy (i.e., a broader range of terms and databases, multiple search approaches) captured a pool of relevant studies not included by our predecessors.

Third, many prior reviews did not take full advantage of emerging meta‐analytic techniques. That is, most reviews did not use advanced approaches such as (a) robust variance estimation to account for within‐study variation and possible issues with effect size dependence or (b) multilevel modeling to account for effect size nesting. Prior reviews also did not test moderation models to examine whether outcomes varied by important features of student training, student characteristics, or examine the impact of SM by subtypes of challenging behavior (i.e., direct and indirect forms of aggression, overt and covert antisocial, and common acts of insubordination). Though two prior reviews did attempt to conduct component analyses of SM and investigate how specific elements were related to effect size estimates (Briesch & Chafouleas, 2009; Fantuzzo & Polite, 1990), those studies did not take advantage of models that may examine whether student participation in each of the SM components impacted outcomes. Because researchers routinely hypothesize that direct student involvement in each SM process would impact the success of the intervention, such analyses would make an important contribution to the current state of research underlying the effects of SM.

Lastly, it is important to consider the overall quality of previous reviews in regard to methodological rigor. That is, findings from previous reviews must be considered and contextualized based on the quality of methodological, logical, and transparent processes utilized. As previously noted, one‐third of the previous reviews conducted in this area did not involve quantitative synthesis, and instead focus on describing SM intervention characteristics, student outcomes, and study features. This indicates a wide variability in terms of quality of review methods and synthesis approaches (i.e., 7 studies focused on descriptive reviews and 14 involved meta‐analysis). Thus, it is our hope that the current study improves upon prior reviews by explicitly and transparently utilizing high‐quality and methodologically rigorous approaches.

## 3 OBJECTIVES

The purpose of the review is to inform practice and policy by evaluating the effectiveness of SM interventions designed to reduce challenging classroom behaviors. The following research questions guide this study:

1. How effective are SM interventions at reducing challenging classroom behavior/increasing positive and prosocial classroom behavior?
2. What does the existing body of literature tell us regarding the state of research on SM interventions? Including:
  a. How rigorously has SM been evaluated?
    i. What types of research designs are most commonly used?
    ii. What are the most common measurement instruments used to assess behavioral change attributed to SM (e.g., observations, standardized instruments)?
    iii. What methods are commonly used to report SM intervention fidelity?
    iv. Do studies report measurement reliability characteristics in the studies (e.g., *α*s, test–retest correlations, *κ*s)?
  b. Do student characteristics moderate the success of SM for behavioral outcomes?
    i. Are the effects of SM moderated by student sex?
    ii. Are the effects of SM moderated by student race/ethnicity?
    iii. Are the effects of SM moderated by student age/grade?
    iv. Are the effects of SM moderated by regular/special education?
  c. Do intervention characteristics moderate the success of SM for behavioral outcomes?
    i. Are the effects of SM moderated by student training?
    ii. Are the effects of SM moderated by length of exposure?
  d. Do behavioral subtypes (i.e., prosocial, disruptive, on‐task, following directions) moderate the success of SM interventions for behavioral outcomes?
  e. Do studies communicate strategies for training students in SM—and if so—do training features (e.g., sequenced skills, active learning modalities, sufficient focus on SM skills) moderate student outcomes?
  f. Does the inherent variation of student participation in each of the 11 SM elements moderate outcomes?
  g. Do studies of behavioral SM strategies examine and report academic outcomes—and if so, what are the average effects of SM strategies for academic outcomes (i.e., achievement, work completion)?
  h. Does the level of program fidelity moderate intervention outcomes?

## 4 METHODS

### 4.1 Criteria for considering studies for this review

#### 4.1.1 Types of studies

Studies were eligible for inclusion regardless of publication status and could include journal articles, books/book chapters, government reports, conference proceedings, theses/dissertations, or unpublished reports (e.g., technical reports). Extensive efforts were made to capture both published studies and gray literature (described in Section 5.2).

To be included, published or unpublished reports had to include two types of designs to answer our research questions. The first included type assessed the effects using multiple group‐design studies (i.e., randomized controlled trials [RCT] and quasi‐experimental designs [QED]). The second included type examined the effects of single‐case design (SCD) studies.

For multiple group‐design studies, to be included studies had to employ a RCT or QED (i.e., nonrandom assignment) that compared groups receiving one or more SM interventions with one or more control groups on one or more qualifying behavioral outcome. Multiple group‐design studies were considered QED if group determination was made by employing methods other than random assignment. Given that we anticipated a small number of RCT studies in this area, we chose to additionally include QED studies. In particular, their inclusion would likely allow us to conduct meaningful meta‐analysis that would not be possible based on RCT studies alone.

Inclusion criteria for SCD studies were guided by the Institute of Education Sciences What Works Clearinghouse (IES‐WWC) standards for studies that *meet evidence standards* and *meet evidence standards with reservations*. We chose these standards because they aid in ruling out threats to internal validity. For SCD studies, each study was evaluated on a case‐by‐case basis using the IES‐WWC Standards Handbook, Version 4.0 (WWC, 2017) for single‐case designs. The standards include the following:

- The independent variable is systematically manipulated in the study, and the researcher must determine when and how independent variable conditions change.
- Each study outcome is measured systematically over time by more than one assessor, and the study collects inter‐assessor agreement on at least 20% of the data points in the baseline and the intervention conditions, and the inter‐assessor agreement must meet minimal thresholds (i.e., **≥**80% if measured by percentage agreement ≥0.60, if measured by Cohen's *κ* [1960]).
- The study includes at least three phases to demonstrate an intervention effect at different points in time (e.g., reversal, multiple baseline).
- Each phase must have an adequate number of data points.
  o For reversal designs, studies must include a minimum of four phases per case with at least five data points per each phase to *meet evidence standards without reservations* or include a minimum of four phases per case with at least three data points per each phase to *meet evidence standards with reservations*.
  o For multiple baseline designs, studies must include a minimum of six phases with at least five data points per each phase to *meet evidence standards without reservations* or include a minimum of six phases with at least three data points per phase to *meet evidence standards with reservations*.

SCD studies that did not *meet evidence standards without reservations* or *meet evidence standards with reservations* were excluded from this review.

#### 4.1.2 Types of participants

To be included, studies had to include students with challenging behaviors who: were of school age (i.e., 5–21 years); of regular or special education status (e.g., emotionally disturbed, learning disabled, other health impaired, etc.); attended an elementary, middle, or secondary school program (i.e., public, alternative, special education, charter, or private school) and presented challenging behaviors. Some studies have suggested that SM is not effective at improving behavioral functioning and may not be feasible for individuals with severe or profound intellectual disability (Ganz & Sigafoos, 2005; Lancioni & O'Reilly, 2001; Shapiro, 1981). Thus, studies including students with severe or profound intellectual disability were not included in this review.

#### 4.1.3 Types of outcome measures

The research underlying the effectiveness of SM suggests the intervention is effective at improving both academic and behavioral outcomes (Briesch & Chafouleas, 2009; Mooney et al., 2005). Thus, the current study included classroom behavior (e.g., disruptive behavior, on‐task, prosocial skills) as the primary outcome of interest, and academic outcomes (e.g., course grades, assignment grades, standardized testing results, work completion) as the secondary outcome of interest.

##### Primary outcomes

The review included only studies that reported outcomes assessing student classroom behaviors—including both challenging and positive classroom behaviors. The following types of challenging behavior outcomes were of interest in this review: (1) aggressive (e.g., hitting or name calling and spreading rumors or betrayal, (2) antisocial (e.g., stealing, bullying, lying, cheating), and (3) insubordinate behaviors (e.g., noncompliance, withdrawal, refusal to cooperate, or off‐task). Further, we were interested in assessing the effects of SM interventions on improving positive classroom behaviors (e.g., on‐task, prosocial skills, following directions). Measures of classroom behavior included standardized measures of challenging and positive classroom behavior. Measures of classroom student behavior were also assessed using daily classroom observational data. That is, teacher observation or third‐party observations of all relevant classroom behavior outcomes (e.g., student off‐ or on‐task behavior, disruptive behavior, positive social interactions).

##### Secondary outcomes

Since SM has been shown to improve academic performance and rates of work completion and accuracy (Carr & Punzo, 1993; Miller et al., 1989; Mooney et al., 2005), we also examined the effects of behavioral SM on academic outcomes for studies reporting those outcomes (i.e., course grades, assignment grades, standardized testing results, work completion, etc.).

#### 4.1.4 Types of settings

For inclusion, studies had to be conducted in a school setting, including public, alternative, charter, private, or special education settings.

#### 4.1.5 Types of intervention

The review included only studies that identified use of a SM intervention, defined as a cognitive behavioral intervention that trains students in a set of techniques necessary to self‐assess, self‐monitor, and self‐evaluate behavioral performance using one or a combination of the following 11 sub‐elements:

1. self‐selecting a target behavior
2. self‐defining the target behavior
3. self‐determining a performance goal
4. self‐identifying reinforcers
5. self‐prompting a reflection of behavior
6. self‐observing a target behavior
7. self‐recording the observations
8. self‐charting the observations
9. self‐appraising performance
10. self‐administering primary reinforcers
11. self‐administering secondary reinforcers

#### 4.1.6 Exclusion criteria

Studies of SM strategies with students who did not present challenging behaviors as the main reason for the intervention were not included in the review (e.g., self‐regulated learning). All searches were limited to studies published since 1988. This year was selected due to its inclusion of the time frame covered in three previous SM reviews (i.e., Briesch & Chafouleas, 2009; Maggin et al., 2013; Mooney et al., 2005) and beginning at the time where the oldest review terminated search procedures (i.e., Fantuzzo & Polite, 1990). In addition, because SM requires the use of multiple metacognitive strategies, SM studies with participants who exhibited severe or profound intellectual disability were excluded. Determination of severe or profound intellectual disability level was made based on author‐provided descriptions or reported intelligence quotient (IQ) scores if no descriptions were provided (i.e., mild = above 55; moderate = 41 to 55; severe = 25 to 40; profound = below 25). Finally, studies were limited to those reported in English due to a lack of availability of interpretation services.

### 4.2 Search methods for identification of studies

**Figure 1:**
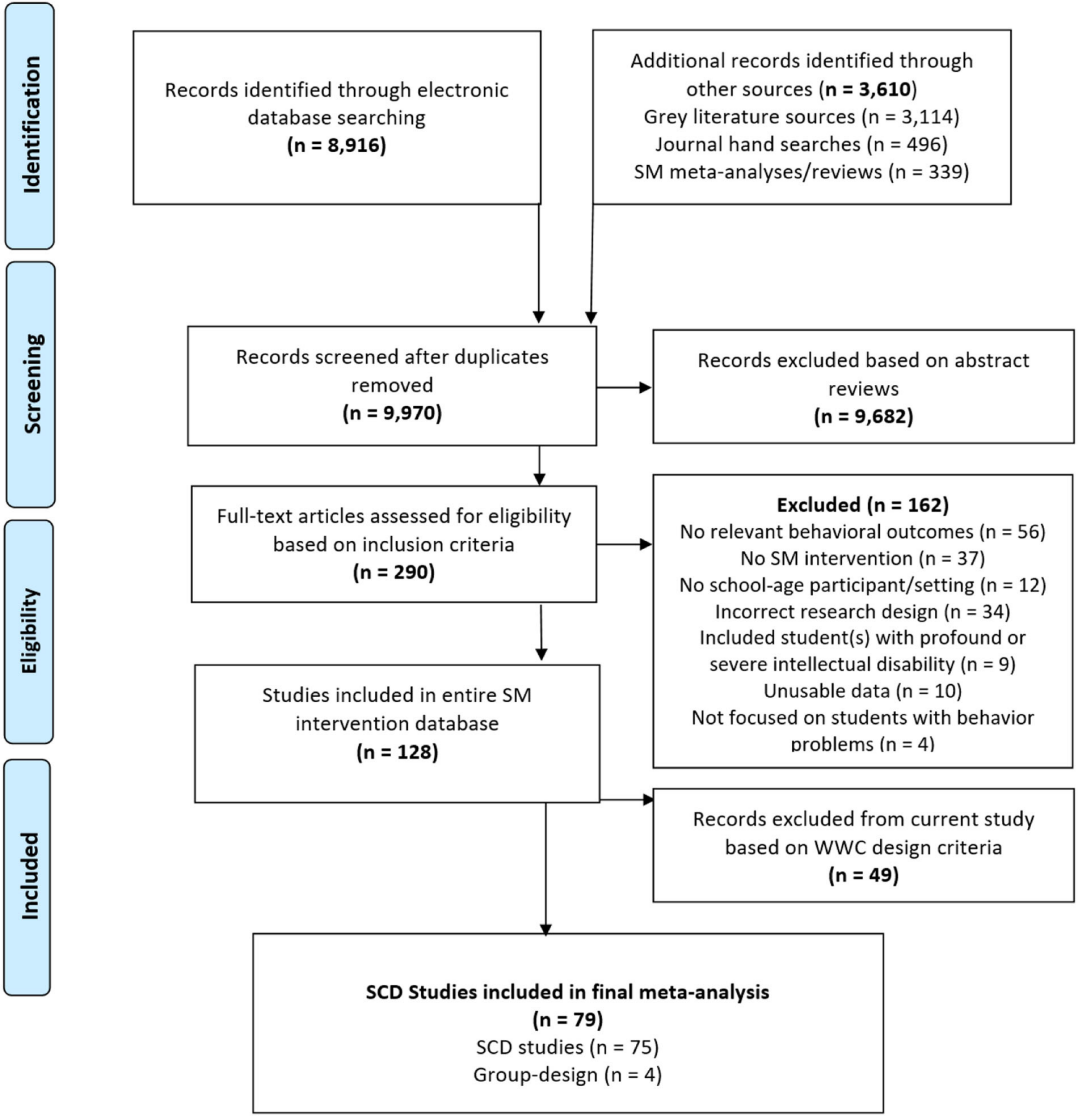
Flowchart of search and screening processes

The following section describes our search procedures for locating potentially relevant studies. All search procedures were conducted by two individuals in August of 2017. The same search procedures were replicated in December of 2020 to screen and ultimately include the most recent relevant literature. To retrieve eligible studies, we utilized several search strategies in an attempt to identify and retain published and unpublished studies. In particular, we electronically searched databases and research registries, took steps to capture gray literature, hand searched relevant journals, and reviewed reference lists of recent reviews of SM interventions. Rationale for selecting electronic databases and other online resources was based on consultation with university library staff, reviewing search methods used in previous relevant meta‐analyses and Campbell Reviews, and our research team's experiences with conducting large‐scale reviews. See Figure 1 for an overview of all search and screening processes and results. All citations yielded from our search methods were logged, saved, and organized using Mendeley management reference software.

#### 4.2.1 Electronic searches

Relevant studies were identified through electronic searches of academic databases and research registries. The following electronic databases and research registries were searched:

1) Electronic databases
  a. Academic Search Premier (EBSCOhost, 1911‐present)
  b. Medline (EBSCOhost, 1946‐present)
  c. APA PsycARTICLES (EBSCOhost, ‐present)
  d. APA PsycINFO (EBSCOhost, 1887‐present)
  e. Social Service Abstracts (ProQuest, 1979‐present)
  f. Sociological Abstracts (ProQuest, 1952‐present)
2) Research registries
  a. Cochrane Collaboration Library
  b. Database of Abstracts of Reviews of Effectiveness
  c. National Technical Information Service

All electronic databases and research registries were originally searched in August of 2017. We updated our search in December 2020 using identical procedures and search strings. Details on our search strings, search limitations, and number of results per electronic database and research registry are presented in Supporting Information Appendix A. Our search strings were created based on consultation with researchers, librarians, and other content experts and aimed to cover aspects of pertinent population, domain, treatment, and outcome information.

#### 4.2.2 Gray literature

Various approaches were also utilized to account for potentially relevant sources of gray literature. In particular, we searched a variety of online resources including additional electronic databases, conference abstracts and proceedings and national government clearinghouses/websites. Further, we attempted to contact authors identified from previously published meta‐analyses and large‐scale reviews. In particular, we searched the following online resources:

1) Electronic databases
  a. ProQuest Dissertation & Theses
  b. Google Scholar
2) Conference abstracts and proceedings
  a. The Society for Research on Educational Effectiveness (SREE)
    i. https://www.sree.org/pages/conferences/index.php
  b. American Educational Research Association Repository (AERA)
    i. https://www.aera.net/Events-Meetings
3) National clearinghouse/government websites
  a. The US Department of Education's web site contains reports of funded programs and initiatives: http://www2.ed.gov/about/offices/list/opepd/ppss/reports.html
  b. The Institution of Education Sciences, What Works Clearinghouse (IES‐WWC) contains reports of intervention investigations: http://ies.ed.gov/funding/grantsearch/index.asp

We aimed to replicate our search strings and parameters based on our electronic search procedures (Section 4.2.1), as this would ensure an unbiased search strategy across all electronic databases and online resources. However, this is challenging in practice due to varying search capabilities across online resources. Thus, searches were slightly altered and simplified to fit the search capabilities of Google Scholar, both conference abstract and proceeding repositories (i.e., SREE, AERA), and for the US Department of Education's website. For instance, Google Scholar does not allow for truncation and limits search strings to 256 characters. Thus, our Google Scholar search string included “self‐monitor OR self‐manage OR self‐record OR self‐evaluate AND class OR child OR school OR student AND behavior OR social OR emotion.”

Lastly, we contacted authors in an attempt to obtain unpublished or ongoing research studies. Authors to be contacted were initially identified based on previously published relevant meta‐analyses and systematic reviews (i.e., Briesch & Chafouleas, 2009; Fantuzzo & Polite, 1990; Mooney et al., 2005). Additionally, throughout our search and review procedures, the first author compiled a list of additional authors to be contacted based on both newly discovered relevant systematic reviews and meta‐analyses and authors/research groups with multiple studies that met our inclusion criteria. These procedures resulted in the first author contacting nine authors. Seven authors responded; however, none had unpublished data to share. One author sent two recently published SM articles; however, these articles had already been identified through other search procedures.

#### 4.2.3 Hand searches of relevant journals

To supplement electronic database searching and gray literature processes, the first author and two trained graduate students hand searched relevant empirical journals known for publishing studies on self‐management interventions. This included searching the following 19 journals spanning across psychology, education, child development, and related fields:

- *Behavioral Disorders*
- *Behavior Modification*
- *Behavioral Interventions*
- *Education and Treatment of Children*
- *Exceptional Children*
- *Child Development*
- *Children & Schools*
- *Journal of Applied Behavior Analysis*
- *Journal of Behavioral Education*
- *Journal of Educational Psychology*
- *Journal of Emotional and Behavioral Disorders*
- *Journal of Positive Behavior Interventions*
- *Journal of School Psychology*
- *Journal of Special Education*
- *Psychology in the Schools*
- *Remedial and Special Education*
- *School Mental Health*
- *School Psychology*
- *School Psychology Review*

Hand search procedures replicated electronic database search processes as closely as possible. However, searches sometimes varied depending on the specific journal searched. For instance, although inclusion criteria ranged from 1988 to 2020, this time period could not be searched for all journals (e.g., *Journal of Emotional of Behavioral Disorders* was not in production until 1993). Further, most journals allowed us to exactly replicate our search strings used during our electronic database procedures via their online database, whereas three journals (i.e., *Children & Schools*, *Education and Treatment of Children*, and *School Mental Health*) did not have the same capabilities. When search strings could not be replicated, simplified search combinations were utilized, followed by screening of titles. For example, *Children & Schools* only allowed us to combine two search strings, as opposed to the four aspects covered by our entire combination of four search strings (i.e., population, domain, treatment, and outcome; see Supporting Information Appendix A). Given the journal's emphasis on school populations and child outcomes, we conducted our search using a combination of the domain and treatment search strings. A total of 496 potential citations were located throughout hand searching procedures.

#### 4.2.4 Reference list searching of previous SM reviews

As a secondary approach to identifying potential studies for inclusion, reference lists of previously published relevant SM meta‐analyses and large‐scale reviews were searched and screened by the first author and two trained graduate students. Twenty‐one reviews were identified in total (see *References to Previously Published SM Reviews*) based on the screening of electronic database results, gray literature sources, and citations identified through hand searches. In total, 339 potential citations were identified based on full reviews of reference lists. Of these, five were included in our final study sample.

### 4.3 Data collection and analysis

#### 4.3.1 Selection of studies

All citations located through searching procedures were imported into Mendeley reference management software (http://www.mendeley.com/). The use of Mendeley allowed us to automatically extract bibliographic data and abstracts from journal articles, book, and/or references. Additionally, we removed all duplicate citations once they were imported into Mendeley.

Next, we selected studies for inclusion based on the following steps—abstract screening and full‐article reviews. First, the first and second author, along with six trained graduate students, independently initially screened abstracts to exclude any studies that were clearly irrelevant. Approximately 41% of all abstracts were double‐screened and compared for inconsistencies during bi‐weekly team meetings. When disagreements occurred, decisions on inclusion/exclusion were determined by the first or second author. Additionally, if abstracts did not provide enough information for inclusion/exclusion, they were included at this stage of the selection process.

The second stage of the selection process involved two research team members independently reviewing the full‐text version of each article identified during abstract reviews as potentially relevant (i.e., not clearly irrelevant). Team members were trained on inclusion criteria (described in Section 4.1.1) by making practice determinations regarding if studies met each of our inclusion criteria. After training, all articles were independently double‐reviewed and included only if all inclusion criteria were met. Full‐text screening questions are noted in Supporting Information Appendix B.

Cohen's *κ* coefficient (Cohen, 1960) was used to calculate inter‐rater reliability across the 41% of abstracts double‐screened and for all full‐text articles reviewed. Cohen's *κ* is computed based on the difference between observed ratings of inclusion/exclusion across studies and the probability of expected agreement due to change. Cohen's *κ* was found to be 0.73 at the abstract review stage, and 0.84 at the full‐text review stage, indicating high levels of inclusion/exclusion agreement between reviewers.

#### 4.3.2 Data extraction and management

Two members of our research team independently coded and extracted relevant data from included studies, with all studies double‐coded to allow for assessment of interrater reliability. Relevant coded data included source descriptions (e.g., report type, how the study was located), study methods (e.g., research design, type of participant assignment), dependent variable/effect size information (e.g., means, standard deviations, how outcomes were assessed), and SM intervention descriptors (e.g., SM components utilized, intervention duration). A coding sheet was first piloted across coders and revised. See Supporting Information Appendix C for a detailed coding scheme utilized for both SCD and group‐design studies. After piloting, our coding sheet was then translated to an online survey system that was created in Qualtrics, a cloud‐based subscription software licensed through the University of Missouri. The use of Qualtrics allowed multiple coders to code studies regardless of location in addition to allowing coded data to be stored in a single online location. Once all coding was completed, extracted data was downloaded from Qualtrics and stored as a Microsoft Excel file and analyzed in R. Further, as calculated at the abstract screening and full‐text review stages of the project, we also calculated Cohen's *κ* for study coding. Results revealed a Cohen's *κ* of 0.79, indicating a high level of agreement among coders.

#### 4.3.3 Assessment of risk of bias in included studies

**Figure 2:**
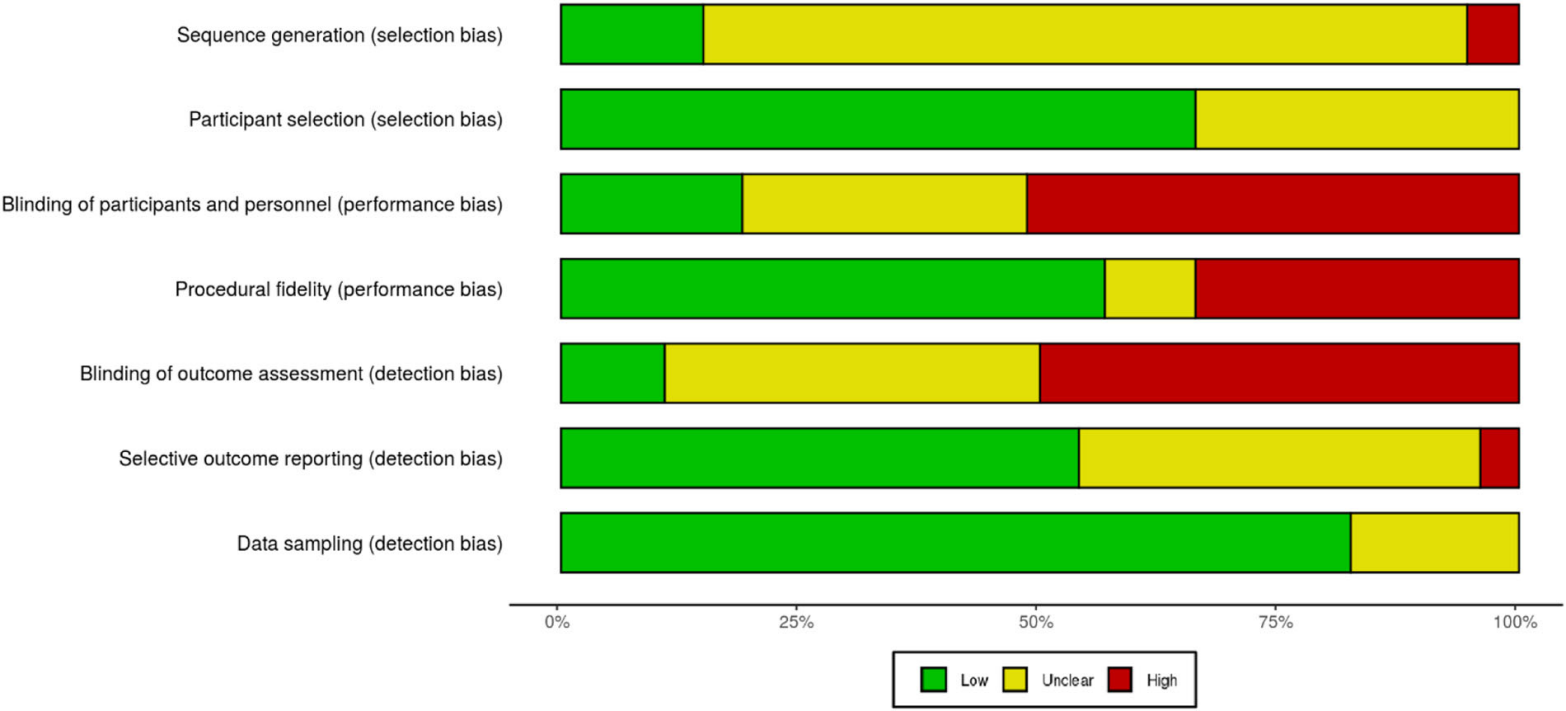
Summary of risk of bias by domain for included SCD studies. SCD, single case designs

To assess risk of bias in included SCD studies, we utilized the Single Case Design Risk of Bias (SCD RoB) tool developed by Reichow et al. (2018). The SCD RoB tool was developed to extend and build upon recent efforts aimed at evaluating methodological dimensions of SCD research to inform causal inferences (Cook et al., 2015; Kratochwill et al., 2013). The SCD RoB tool was conceptualized and modeled off of the Cochrane RoB tool and assesses potential sources of selection bias, performance bias, and detection bias through eight domains (described in Table 3). Compared with other approaches that utilize scoring rubrics and gating processes (Maggin, 2015), the SCD RoB tool utilizes a descriptive framework to document and evaluate potential risk of biases in included studies (Reichow et al., 2018). This descriptive approach allows reviewers to consider risk of bias relative to other pertinent aspects of the review topic without imposing strict scoring processes and/or removing studies all together based on a particular risk factor. Descriptive information is determined based on a review of each domain receiving a code of “low,” “high,” or “unclear” risk of bias. The SCD RoB tool has recently been applied to meta‐analyses including SCD studies across the fields of education (e.g., Martinez et al., 2021), psychology (Davis et al., 2019), and psychiatry (e.g., Im, 2021). Results for our risk of bias assessment for SCD studies are presented in Figure 2.

**Table 3:** SCD RoB domains and descriptions

Type of bias	Domain	Description
Selection bias	Sequence generation	Processes used to allocate participants to intervention conditions or the order of the conditions to which participants are exposed
	Participant selection	Criteria and processes used to include and select participants appropriate for the research
Performance bias	Blinding of participants and study personnel	Procedures used to ensure members of the research team remain unaware of when the intervention is implemented to whom
	Procedural fidelity	Quality of the description for each experimental condition and the reporting of evidence indicating sufficient adherence to the intervention under investigation
Detection bias	Blinding of outcome assessor	Methods used to ensure the individuals collecting outcome data are unaware of the study conditions and research purpose
	Selective outcome reporting	Completeness of the data reported for all participants who began the study including those who withdrew and for each of the dependent variables
	Dependent variable reliability	Methods and reporting of agreement or reliability indices for the outcome variables
	Data sampling	Extent to which the amount of data collected for the research was sufficient to determine the level and trend of the data patterns in each condition to support the determination of a functional relation

Abbreviation: SCD RoB, Single Case Design Risk of Bias.

In the current review, we coded all of the SCD RoB domains with the exception of the “dependent variable reliability” domain. This domain was developed directly based on one of the WWC‐IES design standards. In particular, studies are coded as “low” when mean interobserver agreement is greater than or equal to 80% (or 0.60 for Cohen's *κ*) for all calculations in at least 20% of sessions across phases. This is the same criteria described in the second bulleted WWC‐IES design standard presented in Section 4.1.1. Given that studies had to meet this criterion to be included in our final SCD sample, this domain would have been coded as “low” for all studies. Thus, it did not make sense to additionally code this domain here.

**Figure 3:**
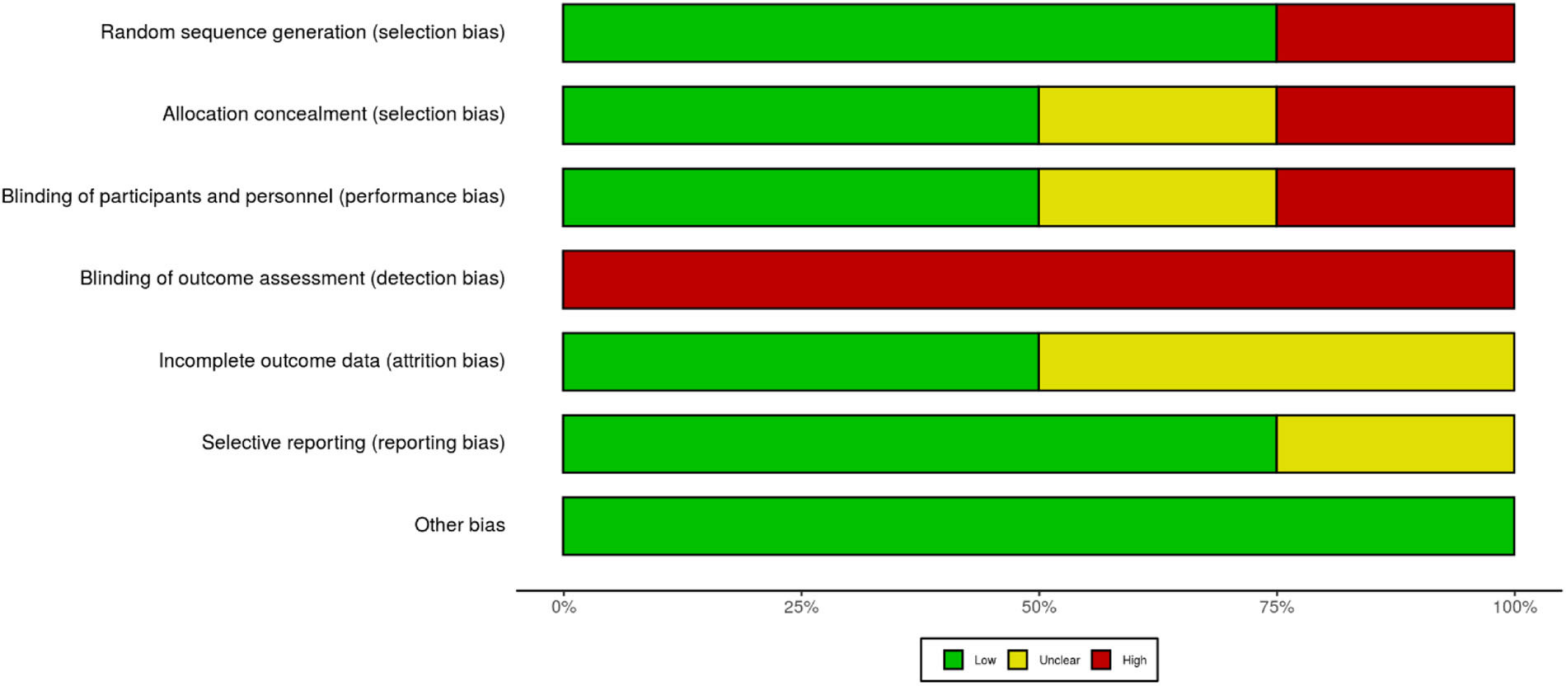
Summary of risk of bias by domain for included group‐design studies

Similarly, we assessed risk of bias for group‐design studies using the Cochrane Collaboration's risk of bias (RoB) tool (Higgins & Altman, Gøtzsche, et al., 2011). In particular, we assessed risk of bias across six domains: sequence generation, allocation, blinding, complete outcome data, selective reporting, and other sources of bias (i.e., deviation from study protocol, inappropriate administration of an intervention, use of an insensitive instrument, and selective reporting of subgroups). The factors assessed within the “other sources of bias” domain were determined based on recommendations from the Cochrane Handbook for Systematic Reviews of Interventions (Higgins & Green, 2011). Each domain was coded as “low,” “high,” or “unclear” risk of bias. Results of the RoB assessment for our four included group‐design studies are presented in Figure 3. For both SCD and group‐design studies, each study was coded independently by two members of our research team, with coders meeting to identify and discuss discrepancies until consensus was met. It is worth noting that the second author of this review was an author for two of the four included group‐design studies. Thus, they did not participate in reviewing risk of bias for those studies.

#### 4.3.4 Measures of treatment effect

Identifying appropriate SCD effect sizes necessary for applying meta‐analytic methods is a consistent challenge and widely debated topic among scholars in this area. Frequently utilized effect size indices (e.g., Percentage of Non‐overlapping Data [PND: Scruggs et al., 1987]; Tau‐U [Parker et al., 2011]) are not well suited for meta‐analysis due to unknown sampling distributions (Shadish et al., 2008) and a lack of comparability across studies in which different measurement procedures are used (Pustejovsky, 2019; Tarlow, 2017). Further, the most frequently used outcomes in SCD studies are behavioral measures collected via systematic direct observations (Ayres & Gast, 2010). Used in conjunction with systematic behavioral observations, scoring procedures, summarizations of scoring (e.g., counts, rates, percentages), and time length of behavior recording can also vary widely across SCD studies.

One effect size index, the log response ratio (LRR; Pustejovsky, 2015, 2018) was recently developed to (a) address limitations noted in commonly used effect indices and (b) serve as a useful means of describing the magnitude of functional relationships for behavioral measures. Further, the LRR is insensitive to procedural variation in how behavioral outcomes are measured and can directly compare behavior assessed based on different dimensional characteristics (Pustejovsky, 2018). Given that the majority of our studies involved systematic direct observations of behavioral outcomes (e.g., on‐task/off‐task behavior, disruptive behavior) using varying scoring procedures (e.g., counts, rates, percentages), we chose the LRR as our effect size index for SCD studies.

For all relevant SCD design cases and outcomes, LRR effect size indices were calculated by extracting raw data from digitized versions of graphs using the data extraction tool WebPlotDigitizer (Rohatgi, 2014). WebPlotDigitizer has previously been shown to yield highly reliable data and indicate a high degree of usability (Moeyaert et al., 2016). All raw data were extracted by the first author and two trained graduate research assistants. LRR indices were then calculated from raw data using an online single‐case effect size calculator (Pustejovsky & Swan, 2018). In particular, we calculated the LRR‐increasing form of the LRR (i.e., the LRRi), so that positive values of effect sizes corresponded to improvement in child behavioral outcomes (e.g., improvements in on‐task behavior, reductions in disruptive behavior).

For group‐design studies, we calculated the magnitude of effect using the standardized mean difference effect size with Hedges' *g* (1981) correction for continuous outcomes. Hedge's *g* effect size index is generally preferred due to its small sample properties. Hedges' *g* was calculated for each relevant effect assessed in our included group‐design study sample. For most group‐design studies, we were able to calculate Hedges' *g* index using means and standard deviations provided by study authors. However, for one study (i.e., Ohakamnu, 2010) we had to calculate Hedges' *g* based on sample sizes and independent sample t‐tests using the following conversion:

$$g=(t)\;\cdot \;\frac{\sqrt{n_{Tx}+n_{Ctl}}}{\left(n_{Tx}\;\cdot \;n_{Ctl}\right)}.$$

#### 4.3.5 Unit of analysis issues

Errors in statistical analyses may occur due to mismatch between the unit of allocation and the unit of analysis. For all SCD studies analyzed here, the unit of analysis was at the individual level. For all group studies, the unit of analysis is at the group level. Across all included studies (both SCD and group‐design), there were no cases in which the unit of assignment did not match the unit of analysis. Additionally, estimates from SCD and group studies were not combined for meta‐analytic purposes. Finally, as most studies yielded multiple effect sizes on the same outcome, data dependency concerns among those nested effect sizes were accounted for through robust variance estimation and multilevel meta‐analysis.

#### 4.3.6 Methods of dealing with dependent effect sizes

In contrast to basic meta‐analytic methods that involve one effect size estimate per study and assume that different studies are independent from one another, LRR effect size estimates describe results at the level of the individual case rather than the study level. Thus, studies that include multiple cases per study contribute multiple effect sizes to the overall meta‐analysis. To account for potential issues with within study dependence and multiple effect sizes per study, we followed guidelines recommended by Pustejovsky (2018) based on a proposed three‐level, hierarchical meta‐analysis model for synthesizing effect size indices from SCD studies (Van den Noortgate & Onghena, 2008). In particular, we applied hierarchical models to synthesize our LRRi effect size estimates, and then utilized robust variance estimation (RVE) techniques (Hedges et al., 2010) to account for potentially inaccurate sampling variances. All RVE procedures were conducted in R using the clubSandwich package (Pustejovsky, 2017; Pustejovsky & Tipson, 2018). We followed a similar approach for group‐design studies. That is, we used RVE to compute pooled effect sizes to account for data dependency issues. In particular, all group‐design studies included more than one measure for the same construct, thus RVE was an appropriate approach. This approach has been utilized recently within educational research involving group‐based interventions within schools (e.g., Sheridan et al., 2019; Smith, Holmes, et al., 2020; Smith, Sheridan, et al., 2020).

#### 4.3.7 Dealing with missing data

For both SCD and group‐design studies, we assessed missing data and attrition rates using risk of bias tools. Both the group‐design and SCD tools allowed us to assess the completeness of the data reported for all included participants who began the study, in addition to accounting for participants that may have withdrawn from the study at any point. In addition, we contacted first authors of group‐design studies in which it was not feasible to estimate effect sizes based on reported data. We also contacted first authors of SCD studies in which graphed data were illegible or appeared to be incorrect (e.g., SCD graphs appeared to be identical for two different participants). When authors did not provide requested data or clarification, we excluded them from the meta‐analysis.

#### 4.3.8 Assessment of heterogeneity

For group‐design studies, we had planned on conducting a test of homogeneity (Q‐test) to compare observed variance to what would be expected from sampling error. We had additionally planned on calculating an *I* ^2^ statistic to describe the percentage of total variation across studies due to the heterogeneity rather than chance. Unfortunately, these proposed assessments of heterogeneity for group‐design studies were not possible given the small number of studies that met inclusion criteria. Additionally, the RVE approach we chose to utilize does not estimate heterogeneity in the same manner as traditional multivariate meta‐analysis. That is, the Q‐statistic and *I* ^2^ statistic are not relevant within the context of RVE. Rather, RVE calculates an overall between‐study heterogeneity that results as a Tau‐squared index that does not include an attendant test statistic or significance test (Tanner‐Smith & Tipton, 2014). We report these values in Table 8 with our group‐design meta‐analysis results. For SCD studies, we calculated and provided interpretations of both case‐level and study‐level variance components. In particular, we calculated variance components *ω* ^2^ (across cases) and *τ* ^2^ (across studies) from our multi‐level meta‐analysis models produced using restricted maximum likelihood methods created using the metaphor package in R (Viechtbauer, 2010). This approach was based on recommendations by Pustejovsky (2018), and has recently been utilized in SCD meta‐analyses assessing the effects of Stay‐Play‐Talk interventions, Social Stories, and peer reporting interventions on child behaviors (Ledford & Pustejovsky, 2021; Wahman, Pustejovsky, Ostrosky, & Milagros Santos, 2019; Collins et al., 2020). We report these values in Tables 7, 9, and 10 with all LRRi effect size meta‐analysis summaries for SCD studies.

#### 4.3.9 Assessment of reporting biases

Meta‐analyses of SCD studies are historically deficient in reporting issues related to publication bias (Gage et al., 2017; Vannest et al., 2018). This is problematic given that there is good reason to suspect that reporting biases may likely occur within the context of SCD studies, especially given the emphasis toward visual analyses for functional relations (Kratochwill et al., 2014). To date, there is no standard or clear recommendations for assessing reporting bias in SCD reviews (Pustejovsky & Ferron, 2017). That said, we attempted to locate and include unpublished findings through our comprehensive literature search procedures that included searching Google Scholar, ProQuest Dissertations & Theses, and contacting authors. Given that our final sample included both published studies and unpublished dissertations/theses, we added analyses to explore if our meta‐analyses findings were moderated by study type (i.e., journal article or dissertation/thesis). Effect sizes did not vary significantly based on study type; thus, we chose to include both study types in all analyses. Unfortunately, there were not enough group‐design studies used to address our research questions to allow us to compare mean effect sizes from journal articles and dissertations/theses in the same way.

#### 4.3.10 Data synthesis

The primary purpose of the current study was to assess the impact of SM intervention on challenging classroom behaviors, in addition to academic achievement and work completion as secondary outcomes. Individual effects were synthesized and analyzed for 75 SCD studies and 4 group‐design studies. In particular, for SCD studies we followed guidelines recommended by Pustejovsky (2018) based on a proposed three‐level, hierarchical meta‐analysis model for synthesizing effect size indices from SCD studies (Van den Noortgate & Onghena, 2008). We applied hierarchical models to synthesize our LRRi effect size estimates, and then RVE techniques to account for potentially inaccurate sampling variances. Specifically, we used cluster‐robust variance estimation methods with small sample adjustments (Tipton & Pustejovsky, 2015) to account for potential inaccuracy of standard errors for individual LRRi estimates, which may occur if autocorrelation or trend is present in the data. We followed a similar approach for our group‐design studies by computing pooled effect sizes with RVE techniques to account for data dependency issues.

For moderation analyses, we utilized separate meta‐regression models for each moderator, pooling across challenging behaviors overall. In particular, we conducted joint tests to assess between‐group effects using the Wald_test function within the clubSandwich R package. This function incorporates a sandwich estimator for the variance‐covariance matrix and applies a small sample correction for estimated *p* values. Further, to conduct SM intervention component analyses, we calculated pooled effect sizes for challenging behaviors (overall) and each of the 11 SM intervention components. It is worth noting that almost all interventions included the use of multiple components. Thus, there were overlaps in the effect sizes used to calculate pooled effect sizes for each SM intervention component. Moderation analyses and component analyses could only be conducted for the SCD studies, given the small number of group‐design studies included in our final sample.

#### 4.3.11 Sensitivity analysis

For group‐design studies, we planned to test the robustness of conclusions drawn from our meta‐analysis through a sensitivity analysis of classroom behavior subtype and type of reporter (e.g., teacher, child). However, we did not have a sufficient number of studies to conduct this analysis. This also excluded us from being able to conduct a “one‐study‐removed” meta‐analysis to determine if results were sensitive to the inclusion/exclusion of particular studies. For SCD studies, to our knowledge, there are currently no clear guidelines or recommendations regarding sensitivity analysis considerations, although it has been recognized as important (Jamshidi et al., 2018; WWC, 2014). For instance, Jamshidi and colleagues (2018) recommend conducting sensitivity analysis within the context of quality assessment and methods utilized within studies. Thus, we chose to conduct a sensitivity analysis based on the two quality codes (i.e., blinding of participants and personnel and blinding of assessors) revealed to indicate high sources of bias across a majority of studies based on our risk of bias assessment. Our meta‐analyses results did not vary significantly with the removal of studies indicating either of these sources of bias. Thus, we chose to keep them in our final sample.

## 5 RESULTS

### 5.1 Results of the search

Throughout our search procedures, we attempted to identify and retrieve both published and unpublished studies that met our inclusion criteria. See Figure 1 for a display of results of our search, screening, and inclusion processes. Our initial electronic search procedures identified 8916 potential records for inclusion. Using other search strategies (i.e., gray literature sources, journal hand searches, and review of SM meta‐analyses/reviews), we identified 3610 other potential records. In all, this resulted in a total of 9970 records after duplicate citations were removed within Mendeley.

#### 5.1.1 Excluded studies

After search procedures were completed, we excluded studies at three different stages: abstract screening, full‐text screening, and reviewing WWC design criteria (for SCD studies only). First, all records were independently screened based on titles and abstracts, of which 4100 (i.e., 41%) were double‐screened. Through the screening process 290 records were identified as potentially relevant.

Next, the full version of these 290 records were all independently double‐reviewed for inclusion based on study inclusion criteria previously described (see Supporting Information Appendix B for full‐text screening questions). Following our published protocol,162 records were removed based on the following reasons: no relevant behavioral outcomes (*n* = 56), no SM interventions (*n* = 37), no school‐age participants/setting (*n* = 12), incorrect research design (*n* = 34), included students with severe or profound intellectual disability (*n* = 9), included unusable data (*n* = 10), or were not focused on students with challenging behaviors (*n* = 4). It is also worth noting that studies could have been excluded for more than one of these reasons. However, we only reported one exclusion reason for each study. See *References to Excluded Studies* for a list of each study excluded throughout this process.

This process resulted in 128 total studies. Of these 128 studies, 4 were group‐design studies, and 124 were SCD studies. Based on our inclusion criteria, we only included SCD studies that met minimum SCD design criteria (i.e., either *meets standards without reservations* or *meets standards with reservations*) based on IES‐WWC guidelines. Therefore, the first author and two trained graduate students independently double‐reviewed each SCD study based on these criteria and excluded an additional 49 SCD studies that did not meet minimum standards. See *References to Excluded Studies*—*Did not meet SCD design criteria based on IES‐WWC guidelines* for a list of each study excluded throughout this process. Thus, our final sample included in our meta‐analysis included 75 SCD studies and 4 group‐design studies.

#### 5.1.2 Included studies

See Figure 1 for an overview of all search and screening processes that led to our final sample of 79 studies. Of these, 75 were SCD and 4 were group‐design. Across 75 SCD studies, our final sample is comprised of 236 participants and 456 effects (i.e., 351 challenging behavior outcomes and 105 academic outcomes). Across the 4 group‐design studies, our final sample included 422 participants and 11 total effects (i.e., 7 prosocial behavior, 2 on‐task behavior, and 2 disruptive behavior). One group‐design study included an academic outcome; however, a single outcome cannot be analyzed in meta‐analyses, and thus was not included in our final sample. Summarized characteristics for the 75 included SCD studies are presented in Tables 4 and 5. Characteristics per each included SCD study are presented in Supporting Information Appendix D. Characteristics of each included group‐design study are presented in Table 6. Further, tables of included studies provide information necessary to answer Research Objectives 2ai, 2aii, 2aiii, and 2aiv (i.e., types of research designs, common measurement instruments/approaches, fidelity methods, and measurement reliability).

**Table 4:** Study, setting, and intervention characteristics of included SCD studies

Study/setting characteristic	*N* (%)	Intervention characteristic	*N* (%)
*Study type* (*n* = 75)		*Measurement instrument/approach* (*n* = 75)	
Journal article	61 (81.33)	Researcher observation	54 (72.00)
Dissertation/thesis	14 (18.67)	Teacher observation	5 (6.67)
		More than one	16 (21.33)
*Search procedures* (*n* = 75)		*Intervention training features* (*n* = 71)	
Electronic search	53 (70.67)	Sequenced training	9 (12.68)
Gray literature	12 (16.00)	Modeling, practice, rehearsal	58 (81.69)
Journal hand search	5 (6.67)	Focused on SM skills (examples/nonexamples)	44 (61.97)
Reference list search	5 (6.67)	Explicit (lessons, manual)	8 (11.27)
*Country* (*n* = 75)		*Intervention fidelity methods* (*n* = 46)	
United States	68 (90.67)	Researcher observations	24 (52.17)
United Kingdom	2 (2.67)	Checklists	11 (23.91)
Canada	2 (2.67)	Participant logs	3 (6.52)
Australia	2 (2.67)	More than one	8 (17.39)
New Zealand	1 (1.33)		
*Community type* (*n* = 49)		*Intervention duration in days* (*n* = 75)	
Urban	30 (61.22)	15 or under	20 (26.67)
Rural	11 (22.45)	16‐30	32 (42.67)
Suburban	8 (16.33)	31‐45	15 (20.00)
		46 or more	8 (10.67)
*School setting* (*n* = 68)		*Intervention components* (*n* = 75)	
Public	51 (75.00)	Self‐assessment	
Alternative	4 (5.88)		
Charter	6 (8.82)	Self‐select target behavior	5 (6.67)
Private	1 (1.47)	Self‐define target behavior	7 (9.33)
Other	6 (8.82)	Self‐determine performance goal[s]	14 (18.67)
		Self‐identify reinforcer	24 (32.00)
*Classroom setting* (*n* = 75)		Self‐monitoring	
Elementary	40 (53.33)	Self‐prompt reflect on target behavior	18 (24.00)
Middle	19 (25.33)	Self‐observe target behavior	70 (93.33)
High	14 (18.67)	Self‐record the observation	71 (94.67)
Mixture	2 (2.67)	Self‐evaluation	
		Self‐chart observations	20 (26.67)
Experimental design (*n* = 75)		Self‐appraise performance	56 (74.67)
Multiple baseline	38 (50.67)	Self‐administer primary reinforcers	5 (6.67)
Withdrawal	32 (42.67)	Self‐administer secondary reinforcers	18 (24.00)
Other	5 (6.67)		

Abbreviation: SCD, single case designs.

**Table 5:** Participant characteristics of included SCD studies

Characteristic	*N* (%)	Characteristic	*N* (%)
Race (*n* = 143)		*Special education status* (*n* = 220)	
Caucasian	76 (53.15)	Yes	137 (62.27)
African American	46 (32.17)	No	83 (37.73)
Latinx	12 (8.39)		
Other	9 (6.29)	*Diagnosis/classification* (*n* = 182)	
		ADHD	78 (42.86)
*Gender* (*n* = 236)		Emotional and behavioral disorder	44 (24.18)
Male	194 (82.20)	Learning deficit	42 (23.08)
Female	42 (17.80)	Autism spectrum disorder	25 (13.74)
		Conduct disorder	9 (4.95)
Grade level (*n* = 236)		Mild or moderate intellectual disability	8 (4.40)
Elementary	135 (57.20)	Speech/language impairment	3 (1.65)
Middle	66 (27.97)	Internalizing concerns	2 (1.10)
High	35 (14.83)		

*Note*: Some students had more than one diagnosis/classification and were captured in more than one category.

Abbreviation: SCD, single case designs.

**Table 6:** Characteristics of included group‐design studies

Study/setting characteristic	Ohakamnu (2010)	Stormont et al. (2020)	Thompson (2014)	Wyman et al. (2010)
Study type	Diss./Thesis	Journal article	Journal article	Journal article
Search procedures	Electronic search	Electronic search	Electronic search	Electronic search
Country	United States	United States	United States	United States
Community type	Urban	Urban	Urban	Urban
School setting	Public	Public	Public	Public
Classroom setting	Elementary	Elementary	Elementary	Elementary
Research design	Quasi‐experimental	Experimental	Experimental	Experimental
Assignment to conditions	Quasi‐random assignment	Random after matching, stratification, or blocking	Random after matching, stratification, or blocking	Random after matching, stratification, or blocking
*Intervention characteristic*				
Measurement instrument/approach	Researcher observation	Standardized instrument	Standardized instrument	Standardized instrument
Measurement reliability	NR	Yes	Yes	Yes
Intervention training features	NR	ST, MPR, SMS, EX	ST, MPR, SMS, EX	ST, MPR, SMS, EX
Intervention fidelity methods	NR	Checklists	Researcher observation	NR
Intervention duration (days)	16‐30	31‐45	31‐45	46 or more
Intervention component(s)	SA4, SM2, SM3	SA1, SA2, SA3, SM1, SM2, SM3, SE1, SE2	SA1, SA2, SA3, SM1, SM2, SM3, SE1, SE2	SA3, SM1, SM2
*Sample characteristics*				
Sample size	51	37	108	226
Race (%)	NR	African American (78); Asian American (0); Caucasian (14); Latinx (5); Other (3)	African American (68); Asian American (1); Caucasian (18); Latinx (7); Other (6)	African American (62); Asian American (0); Caucasian (8); Latinx (26); Other (4)
Gender (%)	Male (39); Female (61)	Male (58); Female (42)	Male (58); Female (42)	Males (54); Female (46)
Grade	5th	4th‐5th	4th‐5th	3rd
Special education (%)	0	100	34	0

*Note*: Intervention training abbreviations: ST = Sequenced training, MPR = Modeling, practice, rehearsal, SMS = Focused on SM skills (examples/non‐examples), EX = Explicit (lessons, manual). Intervention component abbreviations: SA1 = self‐select target behavior, SA2 = Self‐define target behavior, SA3 = Self‐determine performance goal[s], SA4 = Self‐identify reinforcer, SM1 = Self‐prompt reflect on target behavior, SM2 = Self‐observe target behavior, SM3 = Self‐record the observation, SE1 = Self‐chart observations, SE2 = Self‐appraise performance, SE3 = Self‐administer primary reinforcers, SE4 = Self‐administer secondary reinforcers

Abbreviation: NR, not reported.

### 5.2 Description of studies

The following section provides a summary of included studies. First, we provide descriptions of SCD studies organized by (1) study setting and intervention characteristics, and (2) participant characteristics. Next, we provide a description of included group‐design studies, including a table with characteristics for each study. This summary of included studies provides information pertinent to addressing research questions 2ai, 2aii, 2aiii, and 2aiv. Further, results for research question 2aiv are only presented for group‐design studies given that all SCD studies had to report measurement reliability to be included in our study sample.

Table 4 provides a summary of study, setting and intervention characteristics for SCD studies that met WWC design criteria. The majority of included studies came from peer‐reviewed journals (*n* = 61, 81.33%) and 14 (18.67%) were dissertations/theses. Regarding how studies were located, the majority (*n* = 53, 70.67%) came from electronic search procedures, followed by 16.00% via gray literature, 6.67% via journal hand searches, and 6.67% located by reviewing reference lists of previous reviews. The vast majority of studies took place in the United States (*n* = 68, 90.67%), followed by two occurring in each the United Kingdom, Canada, and Australia, and one study taking place in New Zealand. Twenty‐six studies (i.e., 34.67%) did not specify community locale. Of studies that specified locale, 61.22% occurred in urban settings, followed by 22.45% in rural, and 16.33% in suburban. Seven studies (i.e., 9.33%) did not report on school setting characteristics. Of studies reporting school setting, the majority (i.e., 75.00%) were in public schools, followed by 8.82% in charter schools, 8.82% in other school settings, 5.88% in alternative schools, and 1.47% in private schools. Regarding research question 2ai, the majority of included SCD studies used either multiple baseline (i.e., 50.67%) or ABAB (withdrawal) designs (i.e., 42.67%), with 6.67% utilizing other designs (i.e., multiple baseline plus withdrawal, multiple probe, ABABCBC, ABABAC). Regarding research question 2aii, instruments/approaches used to assess measurement effects varied with most studies measuring effects via research observations (i.e., 72.00%), followed by 21.33% utilizing more than one approach (e.g., researcher observation and teacher daily behavior report, teacher observation and student work/academic scores), and 6.67% using teacher observations. Studies additionally varied regarding SM intervention components utilized. That said, nearly all SM interventions included self‐monitoring procedures of self‐observing the target behavior (i.e., 93.33%) and self‐recording of observations (i.e., 94.67%). In contrast, intervention components including self‐selecting a target behavior (i.e., 6.67%), self‐defining a target behavior (9.33%), and self‐administering primary reinforcers (i.e., 6.67%) were infrequently utilized. Regarding research question 2aiii, studies varied in terms of intervention fidelity methods used with the majority using research observation (i.e., 52.17%), followed by 23.91% using checklists, 17.39% using more than one method, and 6.52% using participant logs. All but four (i.e., 5.33%) studies presented information on intervention training features. Of those including intervention training features, studies often included a combination of training features, with the majority (i.e., 81.69%) including modeling, practice, rehearsal, 61.97% focused on SM skills (example/nonexamples), 12.68% sequenced training, and 11.27% explicit lessons or manuals. Twenty‐nine (i.e., 38.67%) of studies did not provide details on intervention fidelity methods. Of studies reporting intervention fidelity, the majority (i.e., 52.17%) used researcher observations, followed by checklists (i.e., 23.91%), more than one method (i.e., 17.39%) or participant logs (i.e., 6.52%). Studies varied in terms of intervention duration with 26.67% taking 15 days or less, 42.67% taking between 16 and 30 days, 20.00% taking between 31 and 45 days, and 10.67% taking 46 or more days.

Table 5 provides a summary of the 236 child participants included in our final SCD sample. Regarding student race, information was reported for 60.59% (*n* = 143) of our sample. Of the participants with race reported, slightly over half were Caucasian (53.15%), followed by 32.17% African American, 8.39% Latinx, and 6.29% Other (multiracial, Middle‐Eastern, Romanian, or Native American). Student gender was reported for all participants. The vast majority of participants were male (*n* = 194, 82.20%) with females comprising less than one‐fifth of the total sample (*n* = 42, 17.80%). Student age was reported for 78.39% (*n* = 185) of our total sample, and indicated an average age of 11.32 (SD = 1.74, range = 5–18). Over half of the sample was comprised of elementary students (*n* = 135, 57.20%), followed by 27.97% (*n* = 66) in middle school, and 14.83% (*n* = 35) in high school. Information about special education status was reported for nearly all of our included sample (*n* = 220, 93.22%), and indicated that the majority (*n* = 137, 62.27%) of participants received special education services in some capacity. Finally, beyond challenging behaviors alone, specific student diagnoses/classifications were provided for the majority (77.12%, *n* = 182) of included participants. Of participants with identified diagnoses or classifications, 42.86% had ADHD, 24.18% were diagnosed with EBD, 23.08% had a learning deficit, 13.74% had ASD, 4.95% were diagnosed with Conduct Disorder, 4.40% had a mild or moderate intellectual disability, 1.65% were classified as having speech/language impairment, and 1.10% had internalizing concerns (e.g., anxiety, depression).

Table 6 includes a breakdown of each of the four group‐design studies that met inclusion criteria with information organized by study, setting, and sample characteristics. One study was a dissertation and the other three were peer‐reviewed journal articles. All studies were located via electronic search procedures. All studies were similar in terms of setting/community characteristics, in that all four studies took place in the United States, were located in urban populations, and focused on elementary populations in public schools. Regarding research question 2ai, all but one study was experimental and used randomization procedures (i.e., randomization after matching, stratification, or blocking). Regarding research question 2aii, one study assessed classroom behaviors via researcher observations, whereas the other three relied on standardized instruments. This differs from SCD studies wherein the vast majority of studies relied on research observations to assess classroom behavior outcomes. Regarding research question 2aiii, two of the four studies did not report intervention fidelity, whereas one study calculated fidelity based on checklists, and one based on researcher observation. Of the three studies that reported intervention training features, all used a combination of all four features. Regarding research question 2aiv, all but one study (i.e., Ohakamnu [2010]) reported reliability of classroom behavior outcomes. Studies varied in terms of intervention duration and intervention components utilized. However, it is worth noting that two studies utilized the same SM intervention (i.e., Stormont et al., 2020; Thompson, 2014). Regarding study participants, three of the four studies were majority male. Of the three studies reporting race, African American students comprised the largest portion of participants across all studies. Finally, studies varied in terms of inclusion of special education populations, with two studies not including special education students, one study solely focused on special education students, and one study including 34% special education students.

#### 5.2.1 Risk of bias in included studies

The following section describes risk of bias summaries for all studies included in our meta‐analyses. We first present results for SCD studies (see Figure 2) followed by group‐design studies (see Figure 3). Figure 2 presents the results for risk of bias for each bias domain for the SCD studies included in our review based on the SCD RoB tool (Reichow et al., 2018). See SupporAppendix E for information on risk of bias for each included SCD study. Results indicated that there was a range of variability across studies. However, risk of bias due to blinding of participants and personnel (n = 38; 50.67%) and blinding of outcome assessment (*n* = 37; 49.33%) were generally high across approximately half of included studies. This indicates that bias may have been introduced due to an inability to conceal research design elements (e.g., which students were receiving the SM intervention) from study participants, personnel, and outcome assessors. Additionally, nearly a third of studies (*n* = 24; 32.00%) did not provide clear documentation regarding procedural fidelity of experimental procedures. This is concerning given that a lack of knowledge regarding the extent to which interventions were implemented as planned introduces substantial risk and should be considered when interpreting results. The majority of studies (*n* = 59; 78.66%) did not provide sufficient information regarding how participants were allocated to intervention conditions. Risk of bias due to data sampling was low in the majority of studies (*n* = 59; 78.66%), indicating that studies included a sufficient amount of data necessary to determine the level and trend of data patterns in each condition to support the determination of a functional relation. Further, the majority of studies (*n* = 49; 65.33%) revealed a low risk of bias based on participant selection, indicating that studies primarily provided clearly elucidated inclusion criteria and showcased that included participants were in need of SM interventions. Only three studies (4.00%) indicated high risk of bias based on selective outcome reporting due to missing data from participants withdrawing from the studies. Finally, only four studies (i.e., 5.33%) indicated a high risk of bias on sequence generation processes used to allocate participants to interventions.

Figure 3 presents the results summary for risk of bias for each of our included group‐design studies. See Supporting Information Appendix F for risk of bias for each included group‐design study. Regarding random sequence generation, only one study utilized nonrandom, quasi‐experimental methods, whereas the other three studies all included detail on randomization and indicated low risk of bias. Results were mixed regarding intervention allocation concealment, with one study indicating selection bias based on allocation due to inadequate concealment, one study not describing allocation concealment in sufficient detail, and two studies describing allocation sequence in sufficient detail. Results were additionally mixed regarding blinding of participants and personnel, whereas one study did not describe procedures in sufficient detail, two studies indicated low risk of bias, and one study notably indicated a high risk of bias due to participants being aware of intervention procedures during the study. Most notably, all studies indicated high risk of bias due to assessors being aware of intervention conditions. Regarding incomplete data and attrition, all four studies either did not describe these details sufficiently or indicated a relatively small amount of attrition (e.g., less than 20% of the study sample). Most studies did not indicate bias due to selective outcome reporting, with one study providing insufficient information to make a judgment. Lastly, none of the four included group‐design studies indicated other potential sources of bias.

### 5.3 Synthesis of results

In the following section we first present results from our SCD studies followed by results from our group‐design studies. It is worth noting that results represent different categorizations of classroom behavior subtypes than what was proposed in our original protocol. Interestingly, no studies included antisocial or aggressive behaviors as defined in our original protocol. Thus, we believed results would be more meaningful with updated categorizations based on behaviors that were assessed within included studies. We provide details and justification for our updated behavior subtype categorizations in our *Deviations from the Protocol* section.

Table 7 represents results of the multi‐level meta‐analysis of LRRi effect size estimates, including estimates of overall average effect sizes, 95% confidence intervals produced from the robust standard errors, study‐level variation, case‐level variation, and corresponding percentage change for classroom behaviors (overall) in addition to each behavior subtype (i.e., prosocial behaviors, on‐task behaviors, disruptive behaviors, and following directions). Table 9 reports the same information for the effects of SM interventions on child academic outcomes (overall) followed by each academic outcome subtype (i.e., academic achievement and work completion). Overall, we synthesized 351 challenging behavior effects across 75 studies and 105 academic effects across 21 studies to estimate the overall effects of SM interventions compared to baseline conditions.

**Table 7:** Behavioral outcomes SCD studies

	*k*	*n*	LRRi (SE)	*CIs*	% change	*t*	Study‐level SD	Case‐level SD
Classroom behaviors (overall)	75	351	0.69 (0.05)	0.59, 0.78	99.37	13.98***	0.16	0.04
Prosocial behaviors^a^	6	19	0.66 (0.17)	0.29, 1.02	93.48	3.84***	0.11	0.07
On‐task behaviors^a^	62	250	0.67 (0.05)	0.57, 0.76	95.42	13.51***	0.13	0.03
Disruptive behaviors^b^	19	61	0.71 (0.16)	0.40, 1.03	51.84	4.42***	0.45	0.01
Following directions^a^	6	24	0.80 (0.17)	0.46, 1.15	122.55	5.07***	0.14	0.02

Abbreviations: CI, 95% confidence interval; *k*, number of studies; LRRi, log response ratio increasing pooled effect size estimate; *n*, number of effect size estimates; SCD, single case designs; SE, standard error.

^a^ Outcome interpreted as increase from baseline levels.

^b^ Outcome interpreted as a decrease from baseline levels.

*** *p* < 0.001.

For group‐design studies, we present results of RVE estimates aimed at accounting for multiple effects reported within a single study. In total, we analyzed the impact of SM interventions across 11 behavioral effects (i.e., 7 prosocial behavior, 2 disruptive behavior, and 2 on‐task behavior). RVE estimates are presented in Table 8. Only one academic outcome was assessed across our four included group‐design studies, and thus, we could not conduct meta‐analysis of group‐design studies for academic outcomes.

**Table 8:** RVE estimates for group‐design studies

	*k*	*n*	Effect size (SE)	*CIs*	*p*	*τ* ^2^	*PIs*
Classroom behaviors (overall)	4	11	0.63 (0.24)	0.08, 1.17	0.03	0.04	0.30, 0.96
Prosocial behaviors	3	7	0.38 (0.07)	0.19, 0.53	0.00	0.01	0.22, 0.54
Disruptive behavior	2	2	0.31 (0.12)	−1.27, 1.89	0.24	0.00	0.24, 0.38
On‐task behaviors	2	2	0.82 (0.62)	−7.04, 8.68	0.41	0.69	−0.41, 1.03

*Note*: CI, 95% confidence intervals; Effect size, RVE pooled effect size estimate (g); *k*, number of studies; *n*, number of effect size estimates; RVE, robust variance estimation; SE, standard error; PIs, 95% prediction intervals.

#### 5.3.1 Meta‐analysis of SM interventions for behavioral outcomes

The following section addresses Research Objective 1 (i.e., the effects of SM interventions at reducing challenging behavior and increasing prosocial behaviors). First, we present results for our SCD studies, followed by results for our group‐design studies. For behavioral outcomes, the overall effects for all five models were significantly different from zero (see Table 7). For classroom behaviors (overall), the average LRRi estimate was 0.69 (95% CI [0.59,0.78]), which corresponds to a 99% change from baseline levels (95% CI [80%, 118%]). For prosocial behaviors, the average LRRi estimate was 0.66 (95% CI [0.29, 1.02]), which corresponds to an increase of 93% from baseline levels (95% CI [34%, 177%]). Regarding on‐task behaviors, the average LRRi estimate was 0.67 (95% CI [0.57,0.76]), which corresponds to an increase of 95% from baseline levels (95% CI [77%, 114%]). For disruptive behaviors, the average LRRi estimate was 0.71 (95% CI [0.40, 1.03]), which corresponds to a reduction of 51% from baseline levels (95% CI [33%, 64%]). For following directions, the average LRRi estimate was 0.80 (95% CI [0.46, 1.15]), which corresponds to an increase of 123% from baseline levels (95% CI [58%, 216%]).

Results of the five models of behavioral outcomes indicate substantially more between‐study variability than within‐study variability in terms of effect sizes. In particular, between‐study SDs for all models ranged from 0.14 to 0.45, indicating substantial heterogeneity in effects across studies. In comparison, within‐study SDs were lower in each model and ranged from 0.01 to 0.07, indicating substantially smaller variance in individual‐specific treatment effects. Assuming normally distributed average effects, results indicated that 67% of effects from future studies should fall between 0.29 and 1.09 for the effects of SM intervention on classroom behaviors (overall). Lower and upper bounds of 67% prediction intervals for each challenging behavior subtype are as follows: prosocial behaviors (0.33, 0.99), on‐task behaviors (0.31, 1.03), disruptive behaviors (0.04, 1.38), and following directions (0.43, 1.17).

For group‐design studies, we present RVE effect size estimates, standard errors, 95% confidence intervals, *τ* ^2^ values, and 95% prediction intervals in Table 8. As previously described, each study included multiple effect sizes on relevant outcome measures. Given that this may result in statistical dependence issues, we conducted RVE estimates to account for shared variation among effect sizes from the same study. Further, positive effect sizes represent mean differences in favor of the treatment group consistent with the intended direction of therapeutic improvement for each outcome. That is, participants receiving SM interventions demonstrated benefits at posttest in comparison to control participants as showcased by greater prosocial skills and on‐task behaviors and lower disruptive behaviors.

For challenging behaviors overall, results indicate a significant and moderate effect of SM interventions on classroom behaviors (*g* = 0.63, 95% CIs = 0.08, 1.17, *p* < .05). For behavior subtypes, we also found significant results for prosocial behaviors (*g* = 0.38, 95% CIs = 0.19, 0.53). No significant results were revealed regarding the impact of SM interventions on disruptive behaviors and on‐task behaviors. However, it is worth noting that both of these effects were only assessed across two effects in two studies.

#### 5.3.2 Meta‐analysis of SM interventions for academic outcomes

The following section addresses Research Objective 2 g (i.e., the effects of SM interventions on academic outcomes). Similar to behavioral outcomes, the overall effects for all three models (i.e., academic outcomes [overall], academic achievement, and work completion) were significantly different from zero (see Table 9). For academic outcomes (overall), the average LRRi estimate was 0.58 (95% CI [0.41, 0.76]), which corresponds to an improvement of 79% from baseline levels (95% CI [45%, 112%]). For academic achievement, the average LRRi estimate was 0.61 (95% CI [0.35, 0.87]), which corresponds to an increase of 84% from baseline levels (95% CI [42%, 139%]). Regarding work completion, the average LRRi estimate was 0.49 (95% CI [0.30, 0.68]), which corresponds to an increase of 63% from baseline levels (95% CI [35%, 97%]).

**Table 9:** Academic outcomes for SCD studies

	*k*	*n*	LRRi (SE)	*CIs*	*%* change	*t*	Study‐ level SD	Case‐level SD
Academic outcomes (overall)	21	105	0.58 (0.09)	0.41, 0.76	78.60	4.67***	0.17	0.03
Academic achievement	13	62	0.61 (0.13)	0.35, 0.87	84.04	5.19***	0.19	0.06
Work completion	11	43	0.49 (0.10)	0.30, 0.68	63.23	5.06***	0.09	0.01

Abbreviations: CIs, 95% confidence intervals; *k*, number of studies; LRRi, log response ratio increasing pooled effect size estimate; *n*, number of effect size estimates; SCD, single case designs; SE, standard error.

*** *p* < 0.001.

Results of the three academic outcome models additionally revealed substantially more between‐study variability than within‐study variability, as evidenced by between‐study SDs ranging from 0.09 to 0.19 (see Table 9). In contrast, within‐study SDs were lower in each model and ranged from 0.01 to 0.06. Assuming normally distributed average effects, results indicated that 67% of effects from future studies should fall between 0.17 and 0.99 for academic outcomes (overall). For academic achievement, 67% of effects should fall between 0.19 and 0.79. Regarding work completion, 67% of effects should fall between 0.17 and 1.05.

Unfortunately, only one academic outcome was assessed across all four of our included group‐design studies. Thus, we could not conduct any analysis of academic outcomes for group‐design studies.

#### 5.3.3 Moderation analyses

All moderation analyses presented in the following section are based on our SCD studies, as our group‐design sample was too small to conduct moderation analyses.

We conducted moderation analyses based on four student characteristics (i.e., age, race, gender, and special education status [Research Objectives 2bi, 2bii, 2biii, and 2biv]) and four intervention characteristics (i.e., student training, duration of intervention, fidelity assessment, and fidelity methods [Research Objectives 2ci, 2cii, and 2h). Research Objective 2h was assessed by determining if results varied based on whether or not fidelity occurred (i.e., fidelity assessment) and/or based on the practices used to assess fidelity (i.e., fidelity method). Further, we had originally proposed to conduct moderation analyses based on both specific training features used to train students in SM (i.e., Research Objective 2e) and each of the 11 SM intervention components (i.e., Research Objective 2f). Nearly all studies included combinations of different training features and different SM intervention components, which did not allow us to isolate potential moderation effects of specific training features or intervention components. Thus, we were unable to conduct moderation analyses for Research Objectives 2e or 2f as hoped (see *Deviations from the Protocol*).

We conducted separate meta‐regression analyses for each potential moderator. Table 10 contains the results of the moderator analyses. Regarding student characteristics, student race (*F* = 5.56, *p* = 0.02) and special education status outcomes (*F* = 6.87, *p* = 0.01) were found to moderate the effects of SM interventions on challenging behavior. In particular, effects were more substantial for African American students compared to other races, and for students receiving special education services in comparison to students who were not. Student age/grade and gender were not found to explain a significant degree in variation of effect size estimates. That said, it is worth noting that effect sizes were higher for both elementary students and male students. None of our four intervention characteristic moderators (i.e., student training, duration of intervention, fidelity assessment, and fidelity method) were found to explain a significant degree of variation in effect size estimates. That said, effect size estimates indicate that effects were higher when intervention procedures included training in SM procedures, lasted for 15 days or less, and did not assess fidelity. Effects also appear to be lower when studies employed more than one method of assessing intervention fidelity (e.g., researcher observations and participant logs).

**Table 10:** Moderation analyses of student and intervention characteristics for behavior outcomes within SCD studies

	*k*	*n*	LRRi (SE)	*CIs*	Study‐ level SD	Case‐level SD	Test of between‐group differences
*Student characteristics*							
Age/grade							*F*(2, 25.1) = 2.31, *p* = 0.12
Elementary	40	190	0.64 (0.08)	0.52, 0.81	0.22	0.04	
Middle	23	120	0.50 (0.07)	0.36, 0.64	0.09	0.01	
High	13	41	0.56 (0.10)	0.36, 0.77	0.11	0.18	
Race/ethnicity							*F*(3, 8.8) = 5.56*, *p* = 0.02
African‐Am.	20	73	0.79 (0.08)	0.58, 0.95	0.09	0.05	
Latinx	10	15	0.53 (0.10)	0.31, 0.74	0.08	0.00	
White	33	128	0.66 (0.07)	0.52, 0.80	0.14	0.03	
Other	6	13	0.65 (0.11)	0.39, 0.91	0.08	0.00	
SPED services							*F*(1, 19.2) = 6.87*, *p* = 0.01
Yes	49	188	0.63 (0.07)	0.47, 0.75	0.22	0.03	
No	27	142	0.49 (0.07)	0.43, 0.69	0.10	0.01	
Gender							*F*(1, 18.3) = 0.02, *p* = 0.90
Female	27	60	0.47 (0.10)	0.28, 0.66	0.23	0.00	
Male	71	190	0.61 (0.04)	0.52, 0.70	0.11	0.03	
*Intervention characteristics*							
Student training							*F*(1, 3.2) = 1.02, *p* = 0.38
Received training	71	340	0.80 (0.16)	0.29, 1.35	0.01	0.05	
No training	4	11	0.63 (0.05)	0.53, 0.73	0.16	0.03	
Duration of intervention (days)							*F*(3, 28.1) = 0.51, *p* = 0.68
15 or less	20	77	0.70 (0.09)	0.52, 0.89	0.15	0.04	
16 to 30	32	153	0.51 (0.09)	0.33, 0.69	0.23	0.04	
31–45	15	70	0.58 (0.07)	0.44, 0.73	0.06	0.01	
46 or more	8	48	0.63 (0.12)	0.39, 0.87	0.09	0.01	
Fidelity assessment							*F*(1, 43.5) = 0.01, *p* = 0.92
Yes	46	219	0.56 (0.06)	0.42, 0.70	0.02	0.21	
No	29	132	0.64 (0.05)	0.53, 0.75	0.05	0.04	
Fidelity method							*F*(3, 7.5) = 4.26, *p* = 0.05
Checklists	11	54	0.64 (0.10)	0.43, 0.84	0.10	0.06	
Researcher obs.	24	143	0.67 (0.07)	0.52, 0.82	0.11	0.07	
Participant logs	3	31	0.55 (0.06)	0.43, 0.68	0.10	0.00	
More than one	8	49	0.43 (0.11)	0.20, 0.66	0.09	0.02	

Abbreviations: CI, 95% confidence interval; *k*, number of studies; LRRi, log response ratio increasing pooled effect size estimate; *n*, number of effect size estimates; SCD, single case designs; SE, standard error; Researcher obs., researcher observations.

* *p* < 0.05.

## 6 DISCUSSION

### 6.1 Summary of main findings

The systematic review and meta‐analysis examined the effects of SM interventions on behavioral and academic outcomes. SM interventions varied a great deal in terms of the specific components and means by which they were implemented. Following the application of systematic search and review procedures, a total of 79 studies were identified which included 75 SCD studies that met rigorous WWC design standards and 4 group‐design studies. In total, the 75 SCD studies examined the effects of SM interventions with 236 K‐12 students on a total of 456 outcomes (351 behavioral outcomes and 105 academic outcomes) while the 4 group‐design studies—3 randomized control studies and 1 quasi‐experimental design—examined the effects of SM interventions with 422 elementary students on 11 total outcomes (7 prosocial behavior outcomes, 2 disruptive behavior outcomes, and 2 on‐task outcomes). On balance, a majority of SCD studies applied SM interventions with male students (82.20%) who were approximately 11.3 years of age. Similarly, about 50% of the students involved in the group‐design studies were male and 3 of the 4 of the group studies were conducted with students in elementary school settings.

### 6.2 Effect of SM interventions for behavioral subtypes

On balance, examining effects from SCD studies revealed that SM interventions appear to be effective at improving student behaviors (LRRi = 0.69) as well as improving academic outcomes (LRRi = 0.58)—corresponding to a 99% and 78% improvement on those outcomes when compared to baseline performance, respectively. More specifically, when examining the effects of SM interventions on specific types of challenging behaviors, SM interventions appear most effective at helping improve student ability to follow directions followed by reductions in disruptive behaviors and increases in prosocial behaviors. When it comes to the effect of SM interventions on specific academic behaviors, SM interventions appear to have the strongest positive effect on helping students complete schoolwork followed by improvements in academic achievement. Group‐design studies, by comparison, suggested moderate effects for improving challenging classroom behaviors (*g* = 0.63) and increasing prosocial behaviors (*g* = 0.38). No effects for academic outcomes were analyzed for group‐design studies as only one study reported an academic outcome.

### 6.3 Interaction of SM interventions with student characteristics

Additional moderation models examined whether the effects of SM interventions varied by student age, race, gender or sex, and special education status. It appears the effects of SM interventions were stronger for African American students compared to Latinx, white, and students identifying as a member of another racial subgroup. That said, this finding needs to be interpreted with caution. For one, Black students appeared to have higher rates of disruptive behavior at baseline, and thus had more room for improvement compared to white students. Further, results may reflect the widely‐known, empirically‐supported, and disparate overidentification and application of behavioral interventions in school settings for youth of color, in particular Black youth. The fact that Black youth make up approximately 13.4% of the population (U.S. Census Bureau, 2020), but represent over 30% of the students in this systematic review of targeted SM behavioral interventions applied in school settings, adds to the mounting evidence of concerns underlying systemic racism and disproportionality in American schools; please note 90.7% of the studies in this review were conducted in US schools. This is not surprising in the context of the persistent and well‐documented achievement gap and stream of studies reporting unequal applications of exclusionary discipline experienced by Black youth (e.g., Cohen et al., 2021). That separate streams of data consistently find disparate outcomes for Black youth in US schools cannot be decoupled from the reality that approximately 79% of teachers in the US education system are White while only approximately 7% of US teachers are Black according to data collected between 2017 and 2018 by the US Department of Education's National Center for Education Statistics (Irwin et al., 2021). The disproportional representation of White and Black teachers in US schools reflect the social, economic, and historical imbalances in the US stemming from slavery and subsequent social and political conditions that persisted thereafter. More specifically, the culture of the majority White teachers in US schools who ultimately define and apply expectations to their students contribute directly, unwittingly or otherwise, to these well‐documented, disparate, and poorer outcomes experienced by Black youth. Ultimately, however, this observation—while concerning—is likely more of an artifact of rating systems and driven by cultural bias rather than any indication that SM interventions are more effective for Black students when compared to white students or students in other racial subcategories (Serpell et al., 2009).

SM interventions also appear to be more effective for students receiving special education services when compared to students in regular education settings. This finding is contrary to a recent meta‐analysis of SM interventions that found special education participants had significantly lower academic engagement (i.e., Bruhn et al., 2020). A trend was noted for SM being more effective for students in elementary settings compared to secondary settings, although this difference was not significant. No differences were noted for boys compared to girls—though most studies reported SM being applied to boys with challenging classroom behaviors. These findings are similar to those examined in a prior systematic review conducted by Bruhn et al. (2020) that noted SM appeared more effective for younger students and for students in special education settings. In practice, behavioral interventions are most often applied to younger students and prior research has suggested these practices are less often applied in middle and high schools (Bruhn et al., 2015; Carter et al., 2011; Mooney et al., 2005; Thompson, 2011; Thompson & Webber, 2010).

### 6.4 SM interventions characteristics

Moderation models were also conducted for four SM intervention characteristics (i.e., student training, intervention duration, fidelity assessment, fidelity method). Surprisingly, while most studies reported providing some level of training (training = 71; no training = 4) it appears that training had little to no effect on behavioral outcomes. This may be an artifact of the reality that there is so little consistency in the manner in which training is provided to students and the literature lacks reporting of any manualized or standardized SM programs from which to examine the effects of standardized training. Further, results did not vary based on intervention duration, whether or not fidelity was assessed, or based on the method used to assess intervention fidelity. That said, it is worth noting that interventions lasting 15 days or less appear to have a slightly stronger effect than interventions of longer duration. Although this should be interpreted with caution due to a lack of significance, this may indicate that even brief SM interventions can have a meaningful impact in improving student challenging classroom behaviors.

### 6.5 Quality of evidence

The overall quality of the evidence suggests that there is a strong bias in the lack of blinding of participants and personnel as well as outcome assessments in the studies included—which is always a concern in SCD. The assessment of bias reflects the incapacity to control or conceal research design elements from raters. Also noteworthy, nearly a third of SCD studies did not provide clear documentation regarding procedural fidelity—making it difficult to clearly understand or document the steps involved and the degree to which these elements were followed. This makes some of the strength of the claims surrounding the elements of SM interventions to be associated with student behavioral outcomes less stable.

### 6.6 Overall completeness and applicability of evidence

The review conducted here is a thorough examination of the existing data on SM interventions for youth who present challenging behaviors in school settings. As prior reviews have noted, SM interventions are one of the most widely used behavioral support interventions in school settings—thus, the findings in this review showcase a wide range of application of this commonly used intervention and attempt to further categorize the various practices to identify the most effective approaches. Using the rigorous criteria guided by WWC‐IES standards to identify the best evidence, our search procedures relied on an exhaustive list of key words related to these intervention procedures as well as rigorous procedures for independent double‐screening and coding of the studies. The result is a comprehensive review of the literature that examines student and context characteristics as well as the use of important training approaches that guide future applications of a widely used and largely effective intervention. This study—like all—does not come without limitations, however, the applicability of the findings should contribute to practices used by school personnel looking to implement effective practices for students with challenging behaviors. It should also be noted that a properly administered SM intervention is also autonomy supportive which is a practice widely accepted to promote student well‐being, responsible decision making, and self‐awareness. In addition, these practices appear to be appropriate and equally effective across elementary, middle and high school conditions.

### 6.7 Limitations and biases in the review process

Although the present study contributes greatly to the field in terms of unmasking elements of a SM intervention that may be more effective, the inability of the review to report these elements with confidence is impacted by the lack of studies included in this review that reported the application of these elements with fidelity ratings. However, the present review does include the use of WWC reporting requirements for SCD—which does strengthen the quality of the evidence collected. Specifically, the 75 SCD studies used in the present review manipulated the independent variable systematically; each study outcome was measured systematically over time by more than one assessor, and the study collected interrater agreement on at least 20% of the data points in both baseline and the intervention conditions; each study included at least three phases to demonstrate an intervention effect at different points in time (e.g., reversal, multiple baseline); and each phase of each study had adequate data.

However, it is clear that the area of SM interventions and the science underlying this effective practice lacks group‐design studies to understand the effect of these practice or the capacity to take these practices to greater scale. This is an area of development of SM interventions and the science supporting SM interventions requires additional study to understand if the elements identified in this study truly relate to better student behavior outcomes.

Another limitation is that we reported Log Response Ratios (LRRi) as an effect size measure of the overall effect of SM on outcomes despite observing trends in the data where certain elements appeared to be differentially effective. Where trends are present in the data the results of LRRi's may produce bias in the ratio estimates (Pustejovsky, 2018).

Further, we excluded studies that involved students with severe or profound intellectual disability. Our justification for this was based on research indicating that SM may not be appropriate for individuals with significant cognitive impairments, as these individuals may have difficulties implementing tasks independently and/or using metacognitive strategies implemented within some SM interventions (Ganz & Sigafoos, 2005; Kahn, 1996). That said, there is also research indicating that SM may be effective for improving academic behaviors (Agran et al., 1989) and social skills (Shukla et al., 1999) for students with severe intellectual disability. Future research in this area should consider including studies involving students with severe and profound intellectual disability.

Another important limitation to note is our decision to test multiple simple meta‐regression models as opposed to a simultaneous meta‐regression model that included all moderators at once. We tested a large number of moderators, and studies typically included some, but not all moderators of interest. The available code used to conduct RVE uses listwise deletion, and thus, multiple meta‐regression would have resulted in a smaller sample to test for moderation effects. With this approach, we erred on the side of using RVE to handle within‐study dependence as opposed to reducing power. That said, our approach of testing a set of simple meta‐regressions does not account for relationships among single moderators in each model and therefore does not assess the unique contributions of each moderator. Future methodological research should consider and work to improve methods for multiple meta‐regression models while handling missing moderators and accounting for within‐study dependence.

In addition, there were many standard limitations that challenge all reviews of this nature, including lack of reported outcomes in some studies, difficulty knowing whether the present study fully represents the universe of SM interventions reported, lack of fully understanding fidelity and elements of each study as well as participant characteristics. As the science in this and other behavioral support interventions moves forward, fully reporting these elements will assist with future reviews to conduct moderator analysis to better understand the most effective elements, which settings these interventions work best in and what types of students and behaviors these practices are most effective for.

### 6.8 Implications for practice and research

Challenging behaviors in school settings are harmful to students and the effective application of practices that guide students to adopt behaviors that are more adaptive is an important responsibility of schools and school personnel. While there are a wide range of universal interventions that appear to be effective at importing useful prosocial skills, there is a documented lack of targeted behavioral support interventions in school settings (Bradshaw, 2015). In addition, Meta‐analyses of targeted behavior support practices reveal that two‐thirds of the few widely used tier 2 behavior support practices to address the needs of students with challenging behaviors are fully managed by adults such as *Check, Connect and Expect* (CCE; Cheney et al., 2009) and *Check‐in Check‐Out* (CICO; Todd et al., 2008) or *The Behavior Education Program* (BEP; Crone et al., 2010). Additionally, not only are there few targeted behavior support interventions available to school practitioners—and that those available are largely adult directed and fails to promote student involvement and autonomy—the effectiveness of those existing and widely used strategies is not supported by data drawn from rigorous research designs or broad bodies of literature and systematic reviews (Bruhn, Lane, & Hirsch, 2014). These issues leave school professionals to address an array of challenging student behaviors with few options; the effectiveness of which is not fully known and the “blanket application” of these widely used tier 2 supports may be inappropriate in some cases. For example, studies of CICO or BEP suggest that when applied to students with escape‐maintained behaviors, teachers are less likely to provide negative feedback to avoid problematic student reactions underlying those behaviors (Reinke et al., 2013). Additionally, developmentally speaking, as youth get into upper elementary and secondary settings, adult managed interventions are at odds with developmental theories as well as the values of youth requiring special education supports where we seek to support psychological and emotional independence and promote self‐determination among students with special needs (Wentzel, 2015). It could be those existing tier 2 strategies (e.g., CICO, CCE, BEP) are optimal for youth motivated by adult attention—though existing research does not examine the effectiveness of these supports with regard to behavioral function. From a development and functional behavior perspective, the blanket application of these widely used tier 2 strategies is simply not always appropriate for some students with EBD. Developmental theory and research suggest that for upper elementary and secondary youth, peer attention and autonomy are more salient needs compared to adult attention; making the approach of existing and widely used tier 2 strategies potentially ineffective and possibly iatrogenic (Reinke et al., 2013). The findings from this review reveal that the use of SM strategies may be an effective means of guiding day‐to‐day implementation of an effective behavior support practice that also supports student autonomy and is in line with developmental theories and the values commonly held in educational settings.

Regarding the implications of this study for advancing research in the area of targeted intervention or intervention development for students with challenging behaviors, the key findings in this study suggest that SM interventions are effective for a range of students, across multiple key behavioral and academic outcomes, and in various contexts. However, the multicomponent nature of included SM interventions made it difficult to disentangle the unique contributions of specific self‐assessment, self‐monitoring, and self‐evaluation components. Moving forward, more research is necessary to determine which SM intervention components, or combinations of components, may be driving beneficial impacts for students. Further, although SM interventions appear to be widely used, it is not known if SM interventions are actually more effective than other widely used teacher‐controlled interventions—or whether the effects of these interventions vary according to student function of behavior. These important research questions—whether SM interventions are more or less effective compared to widely used teacher directed interventions as well as whether either SM or teacher‐directed interventions vary as a function of student behavior are important next steps. Lastly, similar to the variety of ways that SM interventions are implemented, most of the studies employed SCD methodology. To further support the use of SM interventions in schools, more group design studies would help advance our understanding of the effect of SM interventions, particularly with a manualized approach that might incorporate key SM features.

### 6.9 Agreement and disagreements with other studies or reviews

Compared to prior reviews, the present study utilized WWC criteria to identify and include the studies in the present analysis. Only one prior study utilized similar inclusion criteria (Maggin et al., 2013). Comparing the current study to similar recent reviews, the total number of studies netted by review procedures included a range of 30 (Briesch & Chafouleas, 2009) to 66 individual studies (Bruhn et al., 2020). Even after applying rigorous inclusion criteria, the present review netted a total of 79 studies (75 SCD and 5 group). Both prior studies only included SCD studies, but the present review did capture more studies from the existing literature using rigorous inclusion criteria. On balance, the findings of this review largely concur with those of prior reviews regarding the effects of SM interventions in terms of the overall effects on both student behavior and academic outcomes that range from mild to large. The findings from this study also noted little difference across groups of students or settings that SM interventions were used in—including for students with disabilities, by gender or sex, by grade level, or by race. The findings of this review also agreed with the prior observations of Bruhn and colleagues' review (2020) that there were disparate applications of these strategies to youth of color—primarily Black youth compared to their White counterparts. Furthermore, all prior reviews noted the broad range in the means in which SM interventions were implemented in school settings. This review did differ from prior reviews in that our procedures did not note that any studies identified included all 11 components of the original typology used by Fantuzzo (1988) and Briesch and Chafouleas (2009).

### 6.10 Concluding remarks

On balance, the present review adds to the mountain of primary evidence as well as the prior 21 reviews of SM interventions as an effective practice to improve student behaviors in educational settings. The present review found that SM positively impacts both academic and behavioral outcomes for students with challenging behaviors. The present study also presents key findings regarding student and intervention characteristics that influence the impact of SM interventions on important student outcomes.

## ROLES AND RESPONSIBILITIES

Dr. Smith (lead author) was responsible for the overall implementation of the current review beginning in 2017. This included training the research team, organizing resources, helping with search and screening procedures, coding studies, and analyzing data. Dr. Smith also oversaw and helped with tasks such as single‐case data extraction and risk of bias assessment. Dr. Thompson (second author) is the original lead author on the original review protocol. Dr. Thompson was responsible for assisting with overall implementation, and provided help with searching and screening procedures and coding of studies. Dr. Maynard (third author) was also a coauthor on the original review protocol. Given Dr. Maynard's extensive experience with systematic reviews and the Campbell Collaboration, she provided guidance and feedback throughout the process of creating this review.

## DECLARATIONS OF INTEREST

Dr. Thompson is an author on two studies included in this review. Therefore, he did not participate in the coding or risk of bias assessments for those studies. There are no other conflicts of interest to report.

## SOURCES OF SUPPORT

Missouri Prevention Science Institute/Columbia, MO.

## DEVIATIONS FROM THE PROTOCOL

Though we attempted to follow our original protocol (Thompson et al., 2013) as closely as possible, we experienced some circumstances that required us to deviate from our protocol at times. In particular, we deviated from our protocol in terms of our electronic search processes, gray literature searches, overall analytic approaches, challenging behavior definitions, and moderation analyses.

First, regarding our electronic search processes, we originally planned to search Australian Education Index, British Education Index, CBCA Education, and Social Work Abstracts. However, at the times our searches were completed in 2017 and 2020, we no longer had access to these online databases. That said, we added APA PsycARTICLES as an additional electronic search not included in our original proposal. Further, we chose to additionally conduct hand searches of 19 relevant journals (described in Section 4.2.3).

Second, regarding attempts to capture gray literature, we originally proposed to broadly search Google. However, given the large number of potential studies yielded by other methods, we did not feel this was necessary. We also proposed to search the System for Information on Gray Literature, but this no longer existed at the times of our searches.

Third, we deviated from our initial analytic plans. For both group‐design and SCD studies, we initially proposed to calculate effect sizes at the study level. However, since our initial proposal, recent methodological advancements have progressed substantially that account for issues related to effect size dependency when multiple effects are reported per study. In particular, we describe our rationale for using RVE and other recently developed approaches such as multilevel modeling in Section 4.3.6. We additionally deviated in our choice of SCD effect size. In our original proposal, we planned to use a standardized mean difference statistic for single‐case designs developed by Hedges and colleagues (2012). However, given the structure of our data and recently developed SCD effect estimates, we provide a rationale for our decision to instead utilize the LRR effect size index in Section 4.3.4.

Fourth, we ended up changing our categorizations of challenging behavior subtypes based on the outcome characteristics of our study sample. As described previously, we initially proposed to examine challenging behaviors based on the following three subtypes: antisocial, insubordinate, and aggressive. Surprisingly, all challenging behavior outcomes were found to fall into the insubordinate subcategory. That said, acts of insubordination can include a number of behavioral subtypes including noncompliance, withdrawal, refusal to cooperate, impulsivity, inattention, disruptive behavior, and off‐task (Kaiser & Rasminsky, 2009). Further, many included studies would address challenging behavior by attempting to improve desirable, replacement behaviors (e.g., social skills, on‐task behaviors). Thus, we believed it would ultimately be more meaningful and informative to create new categorizations of behavior subtypes using the following four categories: on‐task/off‐task behaviors, prosocial behaviors, disruptive behaviors, and following directions. These categories now capture both challenging and desirable classroom behaviors assessed within included studies. Results are therefore organized based on these distinctions and not the originally proposed subtypes of antisocial, insubordinate, and aggression.

Fifth, we originally proposed to exclude all SM studies with participants who had cognitive impairments or intellectual disability. However, since beginning this review, we have learned that SM interventions appear to be an effective means of improving relevant behavioral outcomes (e.g., on‐task behaviors, prosocial behaviors) for students with mild or moderate intellectual disability. Thus, we decided to only exclude studies including students with severe or profound intellectual disability, as SM interventions may involve multiple, independent, metacognitive strategies that may not be effective or feasible for these individuals (Lancioni & O'Reilly, 2001; Shapiro, 1981). Further, the exclusion of participants with severe or profound intellectual disability has been used by prior relevant meta‐analyses of SM interventions (e.g., Bruhn et al., 2015).

Sixth, in our original protocol, we intended to answer to two sub‐questions under Research Objective 1: (a) Does the use of emerging meta‐analytic techniques for SCDs impact estimated effect sizes compared to prior reviews? and (b) How do efforts to capture all available studies through the use of comprehensive search procedures impact results? These questions were originally intended to address methodological differences and consequences of those differences based on our knowledge of available literature and meta‐analytic techniques established in 2013. Given that we used more advanced meta‐analytic techniques (e.g., RVE) that varied widely from past literature, it no longer made sense to present findings reflective of methodological comparisons and evaluations of their effectiveness in comparison to other studies/approaches. Thus, these two sub‐questions were dropped.

Finally, we had to adjust some of our proposed moderation analyses based on the structure of our data. First, we could not conduct moderation analyses based on SM intervention training features (i.e., sequenced skills, active learning modalities, sufficient focus, and explicit skills) as proposed in Research Objective 2e. However, most studies used a combination of more than one of these features, and thus could not be categorized as distinct groupings to compare to one another. That said, we did do moderation analyses for training overall (i.e., comparing studies that reported training students in SM procedures compared to those that did not), and we report information of training features when describing our study sample in Section 5.2. For the same reason, we could not conduct moderation analyses for each SM intervention component (i.e., Research Objective 2f) because studies used a combination of more than one component. We now note this as a direction for future research.

## Supporting information

Supporting information.
