# Supplementary material for: Self‐management interventions for reducing challenging behaviors among school‐age students: A systematic review
Source: Campbell Syst Rev. 2022 Mar 7;18(1):e1223. doi: 10.1002/cl2.1223 (PMC8902300; doi:10.1002/cl2.1223)
Supplement: Supplementary file 1 — Supporting information. [file CL2-18-e1223-s001.docx]

## APPENDIX A: Electronic Searches

**PART 1 - Electronic database:** EBSCOhost (i.e., Academic Search Premier, Medline, ERIC, APA PsycARTICLES, APA PyscINFO), Social Service Abstracts, Sociological Abstracts

**PART 2 - Research registries:** Cochrane Collaboration Library, Database of Abstracts of Reviews of Effectiveness, National Technical Information System

**Dates:** Original search – August 2017; Updated search – December 2020

**Search limitations:** Language - English only; Date range – January 1988 through August 2017 (original search); September 2017 through December 2020 (updated search)

**Search strings:**

Population – (school* OR class* OR child* OR student* OR adoles* OR teen* OR elementary OR “high school” OR “junior high” OR “middle school” OR “K-12” OR kindergarten)

AND

Domain – (“social behavio*” OR “challenging behavio*” OR “disrupt*” OR “classroom behavio*” OR “student behavio*” OR aggression OR hitting OR “name calling” OR “spreading rumo*” OR antisocial OR stealing OR bully* OR lying OR cheat* OR insubordination OR noncompliance OR withdrawal OR impulsivity OR inattention OR “refusal to cooperate” OR “emotionally disturbed” OR “special education” OR “behavior dis*” OR “problem behavior” OR “emotional dis*” OR “emotional impair*” OR ADHD OR off-task OR on-task)

AND

Treatment – (“self-monitor” OR “self-control” OR “self-report” OR “self-regulat*” OR “self-efficacy” OR “self-prompt” OR “self-record*” OR “self-observ*” OR “self- evaluat*” OR “self-manage*” OR “internal reward*” OR intrinsic OR autonomy OR ownership OR “goal setting” OR “choice making”)

AND

Outcome – (“behavio* competenc*” OR “social competenc*” OR “emotional competenc*” OR “socioemotional skill*” OR “soft skill*” OR “social awareness” OR improve* OR “academic development” OR “academic achievement” OR “academic performance” OR attitud* OR “social development” OR “instructional time” OR “emotional stability” OR grades OR “standardized test*” OR “work completion”)

**PART 1: Electronic database results:**

EBSCOhost* (i.e., Academic Search Premiere, Medline, ERIC, APA PsycARTICLES, APA PyscINFO): 6,940 (original search); 991 (updated search)

Social Service Abstracts: 394 (original search); 19 (updated search)

Sociological Abstracts: 464 (original search); 36 (updated search)

**PART 2: Research registry results:**

Cochrane Collaboration Library -11 reviews (original search); 2 reviews (updated search)

Database of Abstracts of Reviews of Effectiveness – 8 (original search); 11 (updated search)

National Technical Information Service – 40 (original search); 0 (updated search)

*Note: All EBSCOhost databases were searched in combination.

## APPENDIX b: SCREENING QUESTIONS USED AT FULL-TEXT REVIEW

1 . Is the study a Self-Management intervention?

- 1. Yes
- 2. No

2. Does the study focus on student(s) with challenging classroom behaviors?

- Yes
- No

3. Does the study take place in a school setting?

- 1. Yes
- 2. No

4. Does the study examine classroom behavior as an outcome?

- 1. Yes
- 2. No

5. Does the study include school-aged participants?

- 1. Yes
- 2. No

6. Does the study include student(s) with severe or profound intellectual disability?

- 1. Yes
- 2. No

7. Type of research design used within the study:

- 1. RCT
- 2. QED
- 3. SCD
- 4. None of the above

8. Does the study meet inclusion criteria?

- 1. Yes
- 2. If No, why: ___________________________________________ _____________________________________________________

## Appendix C: CODING FORM

**Self-Management Intervention Review Data Coding Form**

Study ID#: __________ Coder: ____________ APA Citation:

**Section A – Source Descriptors & Relevance Screening**

A1. Report Type [type]

- 1. Journal Article
- 2. Book/book chapter
- 3. Gov't report (local, state, federal)
- 4. Conference proceedings
- 5. Thesis or Dissertation
- 6. Unpub report (non-gov’t, tech report)
- 7. Other (specify): ________________

A2. How study was located

- 1. Electronic Database
- 2. Research Registry
- 3. Grey Literature
- 4. Hand Search
- 5. Expert Referral

A3. Country

- 1. USA [coun]
- 2. UK
- 3. Canada
- 4. Australia
- 5. Other (specify): ______________

A4. Setting or region

- 1. Rural [area]
- 2. Suburban
- 3. Urban
- 4. More than one of these settings

A5. Language if other than English _____________ [lang]

**Section B1—Multiple Group (RCT or QED)**

**Study Methods and Quality Determination**

B1.1 Method of assignment to condition(s) [grp_assign]

- 1. Random after matching, stratification, blocking, etc.
- 2. Random, simple
- 3. Quasi-random-assigned by some naturally occurring process
- 4. Matched or statistically controlled on pretest measures
- 5. Does not apply (SCD)
- 99. Not specified

B1.2 Did the study have attrition greater than 20%? [grp_attrit]

- 1. Yes
- 2. No
- 99. Not specified

B1.3 Was there differential attrition in the study?

- 1. Yes
- 2. No
- 99. Not specified

B1.4 If matching was used, how were groups matched? [grp_match]

- 1. Matched on pretest measure
- 2. Matched on demographics
- 3. Matched on both of the above
- 4. Propensity Score Matching
- 5. Other matching technique: ____________________
- 6. Not enough information to determine
- 99. Not specified

B1.6 Was group equivalency at pretest established? [grp_pre]

- - 1. Yes
  - 2. No
  - 99. Not specified

B1.7 Results of statistical comparisons of pretest differences [grp_pre]

- - 1. No comparisons made
  - 2. No statistically significant differences
  - 3. Significant differences judged unimportant by coder
  - 4. Significant differences judged of uncertain importance by coder
  - 5. Significant differences judged important by coder

B1.8 If groups were non-equivalent, were statistical controls used? [grp_ctrl]

- 1. Yes
- 2. No

B1.9 What type of measure was used to assess behavior change? [grp_mstyp]

- 1. Standardized instrument (specify)________________________
- 2. Teacher observation
- 3. Researcher Observation
- 4. Self-report
- 5. More than one of these methods
- 6. Other _________________________
- 99. Not specified

B1.10 Is reliability for Outcome assessment reported? [grp_rel]

- 1. Yes

-Type ________& Level of reliability____________

- 2. No
- 99. Not specified

B1.11 What was the analytical strategy used to assess behavior change? _______________________________________________________________________________________________________________

**Section B2—Multiple Group (RCT, QED)**

**Dependent Variables and Effect Size Information**

B2.1 For RCT or QED designs with continuous dependent outcomes *TX group*

- 1. N _________ [cdv_tx_n]
- 2. Pre-test group mean __________ [cdv_tx_prx]
- 3. Pre-test standard deviation _________ [cdv_tx_sd]
- 4. Post-test group mean________ [cdv_tx_ptx]
- 5. Post-test standard deviation_________ [cdv_tx_sd]

*Comparison Group*

- 5. N _________ [cdv_ct_n]
- 6. Pre-test mean __________ [cdv_ct_prx]
- 7. Pre-test standard deviation _________ [cdv_ct_sd]
- 8. Post-test group mean________ [cdv_ct_ptx]

*Test for Comparing Means & Effect Sizes*

- 9. Are the means adjusted in the analysis? [cdv_adj]
  - 1. Yes (clarify) ____________________________________
  - 2. No
- 10. Statistical value (*t*-test, *F*-value) _______ [cdv_stat]
- 12. Degrees of freedom _______ [cdv_df]
- 13. Reported effect size_____ [cdv_es]

B2.2 For RCT or QED designs with dichotomous outcomes

*TX group*

- 1. N _________ [ddv_tx_n]
- 2. Percent and/or *n* experiencing success _________ [ddv_tx_ps]
- 3. Percent and/or *n* not experiencing success _______ [ddv_tx_pns]

*Comparison Group*

- 5. N _________ [ddv_ct_n]
- 6. Percent and/or *n* experiencing success _________ [ddv_ct_prx]
- 7. Percent and/or *n* not experiencing success _______ [ddv_ct_sd]

*Test for Comparing Means & Effect Sizes*

- 8. Statistical value (*chi2*-test, *F*-value) _______ [ddv_stat]
- 9. Degrees of freedom _______ [ddv_df]
- 10. Reported effect size_____ [ddv_es]

**Section C1—Single Subject (SCD)**

**Study Methods and Quality Determination**

C1.1 The outcomes were measured by more than one assessor [SCD_assess]

- 1. Yes, number of assessors_________________
- 2. No
- 99. Not specified

C1.2 The assessors collected interrater agreement in each phase for 20% of observations (Kappa). [SCD_20p]

- 1. Yes
- 2. No
- 99. Not specified

C1.3 Interrater reliability was =/> .80 for each phase. [SCD_rel]

- 1. Yes
- 2. No
- 99. Not specified

C1.4 The study included more than one phase. [SCD_phase]

- 1. Yes
- 2. No
- 99. Not specified

C1.5 Each phase included at least three observations [SCD_phobs]

- 1. Yes
- 2. No
- 99. Not specified

C1.6 The IV was systematically manipulated by the researcher(s) [SCD_ivman]

- 1. Yes
- 2. No
- 99. Not specified

C1.7 What was the analytical strategy used to assess behavior change? _______________________________________________________________________________________________________________

**Section C2—Single Subject (SCD)**

**Dependent Variable and Effect Size Calculation**

C2.1. What type of measure was used to assess behavior change? [SCD_mstyp]

- 1. Standardized instrument (specify)________________________
- 2. Teacher observation
- 3. Researcher Observation
- 4. Self-report
- 5. More than one of these methods
- 6. Other _________________________
- 99. Not specified

C2.2. Is reliability for Outcome assessment reported? [SCD_rel]

- 1. Yes

-Type ________& Level of reliability____________

- 2. No
- 99. Not specified

C2.3. What was the analytical strategy used to assess behavior change? ________________________________________________________________________________________________________________

C2.4 For SCD-within group designs with continuous outcomes

- 1. N _________ [SCD_n ]
- 2. Pre-test mean __________ [SCD_pre ]
- 3. Pre-test standard deviation _________ [SCD_presd]
- 4. Post-test mean________ [SCD_pt ]
- 5. Post-test standard deviation_________ [SCD_ptsd ]

*Test for Comparing Means & Effect Sizes*

- 6. Statistical value (*t*-test, *F*-value) _______ [SCD_stat]
- 7. Degrees of freedom _______ [SCD_df]
- 8. Reported effect size_____ [SCD_es]

C2.5. For SCD with dichotomous outcomes

*TX group*

- 1. N _________ [SCD_tx_n]
- 2. Percent and/or *n* experiencing success _________ [SCD_tx_ps]
- 3. Percent and/or *n* not experiencing success _______ [SCD_tx_pns]

*Comparison Group*

- 5. N _________ [SCD_ct_n]
- 6. Percent and/or *n* experiencing success _________ [SCD_ct_prx]
- 7. Percent and/or *n* not experiencing success _______ [SCD_ct_sd]

*Test for Comparing Means & Effect Sizes*

- 8. Statistical value (*chi2*-test, *F*-value) _______ [SCD_stat]
- 9. Degrees of freedom _______ [SCD_df]
- 10. Reported effect size_____ [SCD_es]

C2.6. For SCD with multiple baselines,

- 1. Case Id_________ [SCD_ID]
- 2. Number of phase (A1, B1, A2, B2, etc.) [SCD_ph ]
- 3. Number of obs/phase____ [ss_obs/ses]

**D—Participants, Intervention Agents, and Setting Descriptors**

D1. Average Age of participants ___________. [age]

D2. Average grade of participants__________. [grd]

D3. Race/Ethnicity [raceth]

- 1. African American ___%
- 2. Asian American___%
- 3. European American ___%
- 4. Hispanic American___%
- 5. Other ___%
- 99. Not specified

D4. Sex [sex]

- Male___%
- Female___%

D5. Free or Reduced Lunch [frl]

- 1. Receiving ___%
- 2. Not Specified

D7. Setting [schtyp]

- 1. School
- 2. Public
- 3. Private
- 4. Alternative
- 5. Charter
- 6. Other (specify): __________
- 99. Not specified

D8. School [sch_cntr]

- 1. Elementary
- 2. Middle or Junior
- 3. Secondary
- 4. Mixture_________________
- 99. Not specified

D9. Primary education status/eligibility [edstat]

- 1. Regular
- 2. 504 Plan
- 3. Special
  - 4. LD
  - 5. ED
  - 6. Other Health Impaired
  - 7. Other
  - 8. Mixture (cat. & %)____________________
- 99. Not specified

D10. Who provided the services? [intagt]

- 1. Non-School Master’s or PhD clinician
- 2. School Clinician (Social Worker, Psychologist, Counselor)
- 3. Teacher
- 4. Other school personnel
- 5. Researchers (efficacy trial)
- 6. Multiple providers ____________________(list)
- 7. Other: __________________(list)
- 99. Not specified

D11. Did the provider receive special training on the intervention? [tr_intagt]

- 1. Yes
- 2. No
- 99. Not Specified

D12. Subtypes of challenging behavior [behstyp]

- 1. Aggression (direct or indirect physical or social aggression)
- 2. Antisocial (overt or covert)
- 3. Insubordination (noncompliance, withdrawal, refusal, off-task)
- 99. Not specified

**Section E - Intervention Descriptors**

E1. Name of intervention: ______________________________

E2. Did students engage in self-assessment? [int_sa]

- 1. Self-select target behavior
- 2. Self-define target behavior
- 3. Self-determine performance goal[s]
- 4. Self-identify reinforcers

E3. Did students engage in self-monitoring? [int_sm]

- - 1. Self-prompt reflect on target behavior
  - 2. Self-observe target behavior
  - 3. Self-record the observation

E4. Did students engage in self-evaluation? [int_se]

- - 1. Self-chart observations
  - 2. Self-appraise performance
  - 3. Self-administer primary reinforcers
  - 4. Self-administer secondary reinforcers
- 5. Other steps _________________
- 99. Not specified

E5. Did teacher monitor behavior? [int_tchmon]

- 1. Yes
- 2. No_____________________________(list)
- 99. Not specified

E6. What source did students use to compare SM data? [int_tchcom]

- 1. Teacher
- 2. Self-goal
- 3. Prior performance
- 4. Other third party
- 5. Multiple sources: __________________(list)
- 99. Not specified

E7. Were students provided training? [int_trn]

- 1. Yes
- 2. No
- 99. Not specified

E8. Length of training [int_lgth]

- Total number of minutes: _____
- 99. Not specified

E9. Teaching modalities used in training [int_mod]

- 1. Sequenced training (vertical alignment of skills)
- 2. Activities used in training (modeling, practice, rehersal)
- 3. Focused on SM skills (examples/nonexamples)
- 4. Explicit (lessons, manual)
- 99. Not specified

E10. Length of self-monitoring [int_lgth]

- # of weeks: _____
- 99. Not specified

E11. Focus of goal for behavior intervention [int_assess]

- 1. Increase display of positive behavior
- 2. Reduce display of challenging behavior
- 3. Increase positive and reduce challenging behaviors
- 4. Academic performance
- 5. Both behavior and academic outcomes
- 6. Other__________________________

E12. Manner of prompt used in the study? [int_prpt]

- 1. Internal prompt (self-prompt)
- 2. External prompt (third party verbal prompt)
- 3. Technology (assistive device; recording or digital)
- 4. Combination of internal and external prompt
- 99. Not Clear

E13. Type of recording device [int_redev]

- 1. Digital device only
- 2. Paper-and-pencil only
- 3. Assistive device and paper and pencil
- 4. Other __________________________
- 99. Not clear

E14. Behavioral Goal [int_goal]

- 1. Positively worded (presence, positive replacement behavior)
- 2. Negatively worded (absence, cease a challenging behavior)
- 3. Positively and negatively worded goals
- 4. Focus on off/on task behavior
- 99. Not clear

E15. Number of intervals on recording device_____ [int_interval]

E16. Number of recording device response options [int_resopt]

- 1. One
- 2. Tw0
- 3. Three
- 4. Four
- 5. Five
- 6. Six or more
- 99. Not clear

E17. Number of self-evaluation sessions [int_seses}

- 1. One
- 2. Tw0
- 3. Three
- 4. Four
- 5. Five
- 6. Six or more
- 99. Not clear

E18. What type of reinforcement used along with SM? [int_reinf]

- 1. None
- 2. Positive reinforcement
- 3. Negative reinforcement
- 4. Both positive and negative reinforcements
- 99. Not clear

E19. Was the intervention faded? [int_fad]

- 1. Yes
- 2. No
- 99. Not specified

**Section F—Intervention Fidelity**

F1. Did the study report fidelity? [fidel]

- 1. Yes
- 2. No
- 99. Not specified

F2. How was fidelity assessed? [fidel_asses]

- 1. Researcher observations
- 2. Interviews of participants
- 3. Surveys of participants
- 4. Participant logs
- 5. Administrative records
- 6. Checklists
- 7. Other ______________________
- 99. Not specified

F3. Level of adherence to the tx: [fidel_ad]

- 1. Percent or Level ________________
- 99. Not specified

F4. What type of fidelity measures were collected [fidel_typ]

- 1. Structural (count of services delivered)
- 2. Procedural (quality of services delivered)
- 3. Both structural and procedural
- 99. Not specified

| APPENDIX D: STUDY, SETTING, AND INTERVENTION CHARACTERISTICS FOR EACH SCD STUDY | | | | | | | | | | | | | |
| --- | --- | --- | --- | --- | --- | --- | --- | --- | --- | --- | --- | --- | --- |
| **First author (year)** | **Study type** | **Search procedures** | **Country** | **Comm. type** | **School setting** | **Class setting** | **Experi-mental design** | **Meas. approach** | **Int. training features** | **Int. fidelity methods** | **Int. dura-**  **tion (days)** | **SM int. component(s)** |  |
| Aljadeff-Abergel (2011) | Thesis/ Dissertation | Grey literature | USA | Urban | Charter | Elem. | MBL | Researcher obs. | MPR, SMS | Checklists | <15 | SM2, SM3. SE2. SE4 |  |
| Baker (1996) | Thesis/ Dissertation | Grey literature | USA | Urban | Public | Elem. | ABAB | Researcher obs. | SMS | NR | 31-45 | SA1, SA2, SA3, SA4, SM2, SM3, SE2 |  |
| Barry (2003) | Journal article | Electronic search | USA | NR | Public | Elem. | Other | Teacher obs. | MPR, EX | Participant logs | 46+ | SA1, SA2, SA4, SM2, SM3, SE1 |  |
| Beckman (2019) | Journal article | Electronic search | USA | NR | Public | Elem. | ABAB | More than one | MPR, SMS | NR | <15 | SA4, SM2, SM3, SE2, SE3 |  |
| Bialas (2010) | Journal article | Electronic search | USA | Rural | Public | Elem. | MBL | Teacher obs. | MPR | NR | 16-30 | SA4. SM2, SM3, SE4 |  |
| Boswell (2013) | Journal article | Electronic search | Canada | Rural | Public | Middle | ABAB | Researcher obs. | MPR, SMS, EX | More than one | <15 | SA4. SM2, SM3, SE2 |  |
| Briesch (2013) | Journal article | Electronic search | USA | Urban | Charter | Middle | MBL | Researcher obs. | MPR, SMS, EX | Participant logs | 16-30 | SA4. SM2, SM3, SE2, SE4 |  |
| Bruhn (2012) | Journal article | Electronic search | USA | Rural | Public | Mixture | ABAB | Researcher obs. | SMS | Researcher obs. | <15 | SA4. SM2, SM3, SE2, SE4 |  |
| Bruhn (2015) | Journal article | Electronic search | USA | Urban | Public | Middle | ABAB | Researcher obs. | MPR | Researcher obs. | 16-30 | SA4. SM2, SM3, SE2, SE4 |  |
| Bruhn (2016) | Journal article | Electronic search | USA | NR | NR | Middle | ABAB | Researcher obs. | MPR, SMS | Researcher obs. | <15 | SA4. SM2, SM3, SE2, SE4 |  |
| Bulla (2017) | Journal article | Electronic search | USA | Rural | Public | Middle | ABAB | Researcher obs. | SMS | Researcher obs. | 16-30 | SM2, SM3, SE2 |  |
| Caldwell (2010) | Thesis/ Dissertation | Grey literature | USA | Urban | Other | High | ABAB | Researcher obs. | MPR, SMS | Checklists | 16-30 | SA3, SA4, SM1, SM2, SM3, SE1, SE2 |  |
| Callicott (2003) | Journal article | Electronic search | USA | Rural | Alt. | Mixture | ABAB | Researcher obs. | MPR | Researcher obs. | 16-30 | SA4, SM1, SM2, SE2, SE3 |  |
| Christensen (2007) | Journal article | Electronic search | USA | Urban | NR | Elem. | ABAB | Teacher obs. | MPR, EX | Checklists | 31-45 | SM2, SM3 |  |
| Cihak (2010) | Journal article | Electronic search | USA | NR | Public | Middle | MBL | More than one | MPR | Researcher obs. | 31-45 | SM2, SM3 |  |
| Clemons (2016) | Journal article | Electronic search | USA | Rural | Public | High | ABAB | Researcher obs. | MPR, SMS, EX | More than one | <15 | SM2, SM3 |  |
| Coogan (2003) | Thesis/ Dissertation | Grey literature | USA | Urban | Public | Middle | ABAB | Researcher obs. | ST, SMS | Researcher obs. | 46+ | SM1, SM3, SE1 |  |
| Cook (2020) | Journal article | Electronic search | USA | Rural | Public | High | Other | Researcher obs. | ST, MPR | Researcher obs. | 31-45 | SM2, SM3, SE1 |  |
| Coughlin (2012) | Journal article | Electronic search | USA | NR | NR | Elem. | MBL | More than one | MPR, EX | NR | 16-30 | SM1, SM2, SM3, SE1, SE2, SE3 |  |
| Creel (2006) | Journal article | Electronic search | USA | Suburb. | Public | Middle | MBL | Teacher obs. | NR | NR | 16-30 | SM1, SM2, SM3, SE2 |  |
| Crutchfield (2015) | Journal article | Electronic search | USA | Urban | Public | Middle | MBL | Researcher obs. | MPR | Researcher obs. | 31-45 | SA2, SM2, SM3, SE2 |  |
| Dalton (1999) | Journal article | Electronic search | USA | NR | Public | Middle | MBL | More than one | MPR, SMS | NR | 16-30 | SA3, SM1, SM2, SM3, SE2, SE4 |  |
| Davies (2000) | Journal article | Electronic search | USA | Urban | Public | Elem. | ABAB | Researcher obs. | MPR, SMS | NR | 16-30 | SA3, SA4, SM1, SM2, SM3, SE1, SE2 |  |
| Davis (2014) | Journal article | Reference list search | USA | NR | Charter | High | MBL | Researcher obs. | MPR | More than one | 16-30 | SA3, SA4, SM2, SM3, SE2 |  |
| Estrapala (2020) | Thesis/ Dissertation | Grey literature | USA | NR | Public | High | MBL | Researcher obs. | MPR, SMS, EX | More than one | 16-30 | SA1, SA2, SA3, SM1, SM2, SE2 |  |
| Fairfield (2016) | Thesis/ Dissertation | Electronic search | USA | Rural | Public | Elem. | MBL | More than one | ST, SMS | Researcher obs. | 46+ | SM2, SM3, SE1, SE2 |  |
| Fessler (1991) | Thesis/ Dissertation | Grey literature | USA | Urban | Alt. | Elem. | MBL | More than one | MPR | NR | 16-30 | SA3, SA4, SM1, SM2, SM3, SE2, SE4 |  |
| Gureasko-Moore (2006) | Journal article | Electronic search | USA | Urban | Public | Middle | MBL | Researcher obs. | SMS | Researcher obs. | 16-30 | SA2, SA3, SM2, SM3, SE1, SE2 |  |
| Gureasko-Moore (2007) | Journal article | Electronic search | USA | NR | Public | Middle | MBL | Teacher obs. | ST, MPR | More than one | 31-45 | SM1, SM2, SM3, SE1, SE2 |  |
| Hansen (2014) | Journal article | Electronic search | USA | Urban | Other | Elem. | Other | Researcher obs. | MPR | Researcher obs. | 46+ | SA3, SM1, SM2, SM3, SE1, SE2 |  |
| Harris (1994) | Journal article | Electronic search | USA | Suburb. | NR | Elem. | MBL | Researcher obs. | MPR | NR | 31-45 | SM2, SM3, SE1, SE2 |  |
| Harris (2005) | Journal article | Electronic search | USA | Suburb. | NR | Elem. | MBL | More than one | SMS | NR | <15 | SA1, SM3, SE1 |  |
| Hoff (1998) | Journal article | Electronic search | USA | NR | Public | Elem. | MBL | Researcher obs. | SMS | Researcher obs. | 31-45 | SA4, SM2, SM3, SE2, SE4 |  |
| Houghton (1989) | Journal article | Electronic search | UK | NR | Public | High | Other | Researcher obs. | NR | NR | 16-30 | SM2, SM3, SE2, SE4 |  |
| Hutchinson (2000) | Journal article | Electronic search | USA | Urban | Public | Elem. | ABAB | Researcher obs. | MPR | NR | <15 | SA4, SM2, SM3, SE2, SE4 |  |
| Imasaka (2019) | Journal article | Hand search | Australia | Urban | Private | Elem. | MBL | Researcher obs. | MPR, SMS | Researcher obs. | 16-30 | SA3, SA4, SM2, SM3, SE1, SE2, SE4 |  |
| Kern (1994) | Journal article | Electronic search | USA | NR | Public | Elem. | MBL | Researcher obs. | ST, SMS | NR | 31-45 | SM2, SM3, SE2 |  |
| King (2014) | Journal article | Electronic search | USA | Suburb. | NR | Elem. | Other | Researcher obs. | MPR, SMS | Checklists | <15 | SM2, SM3, SE2 |  |
| King (2017) | Journal article | Hand search | USA | Suburb. | Public | Elem. | MBL | Researcher obs. | MPR, SMS | Checklists | 16-30 | SM2, SM3, SE1, SE2 |  |
| Kolbenschlag (2019) | Journal article | Electronic search | USA | NR | Public | Elem. | MBL | Researcher obs. | MPR, SMS | Checklists | 16-30 | SA4, SM2, SM3, SE2 |  |
| Kumm (2019) | Thesis/ Dissertation | Grey literature | USA | Urban | Public | High | ABAB | Researcher obs. | MPR, SMS | Researcher obs. | <15 | SM2, SM3, SE1, SE2 |  |
| Kunsch (2010) | Thesis/ Dissertation | Grey literature | USA | Urban | Public | Middle | ABAB | Researcher obs. | MPR, SMS | Researcher obs. | 16-30 | SM2, SM3, SE2 |  |
| Legge (2010) | Journal article | Electronic search | USA | Rural | Public | Elem. | MBL | Researcher obs. | ST, MPR | Researcher obs. | 31-45 | SM2, SM3 |  |
| Levendoski (2000) | Journal article | Electronic search | USA | NR | Other | Elem. | ABAB | More than one | NR | NR | 46+ | SA3, SM2, SM3, SE1, SE2 |  |
| Lively (2019) | Journal article | Electronic search | USA | NR | Other | High | MBL | Researcher obs. | MPR, SMS | NR | 16-30 | SM2, SM3, SE2 |  |
| Lloyd (1989) | Journal article | Electronic search | USA | NR | Public | Elem. | MBL | Researcher obs. | MPR, SMS | Participant logs | 16-30 | SA1, SM2, SM3, SE2 |  |
| Lo (2006) | Journal article | Electronic search | USA | Urban | Public | Elem. | MBL | Researcher obs. | ST, MPR, SMS | Checklists | 31-45 | SA4, SM2, SM3, SE2, SE4 |  |
| Marshall (1993) | Journal article | Electronic search | USA | NR | Other | Elem. | MBL | Researcher obs. | MPR, SMS | NR | 16-30 | SM2, SM3, SE2 |  |
| Mathes (1997) | Journal article | Reference list search | USA | NR | Public | Elem. | MBL | Researcher obs. | MPR, SMS | NR | 31-45 | SM2, SM3, SE1, SE2 |  |
| McDougall (1995) | Journal article | Electronic search | USA | NR | Public | Elem. | MBL | More than one | MPR, SMS | NR | 16-30 | SM2, SM3 |  |
| McGoey (2007) | Journal article | Reference list search | USA | NR | Public | Elem. | ABAB | Researcher obs. | NR | Checklists | 16-30 | SA2, SM2, SM3 |  |
| Moore (2001) | Journal article | Electronic search | New Zealand | Suburb. | Public | Elem. | MBL | Researcher obs. | MPR, SMS | NR | <15 | SA3, SM1, SM3, SE2 |  |
| Moore (2013) | Journal article | Hand search | Australia | Suburb. | Public | High | MBL | Researcher obs. | MPR, SMS | NR | <15 | SM2, SM3, SE1, SE2 |  |
| Ness (2013) | Journal article | Electronic search | USA | Urban | Public | Middle | MBL | Researcher obs. | MPR, SMS | Researcher obs. | 46+ | SA1, SM2, SM3, SE2 |  |
| O'Reilly (2002) | Journal article | Electronic search | UK | Urban | Public | Middle | ABAB | Researcher obs. | MPR | NR | 16-30 | SA4, SM2, SM3, SE2 |  |
| Peterson (2006) | Journal article | Electronic search | USA | Urban | Public | Middle | MBL | Researcher obs. | MPR, SMS | More than one | 46+ | SM1, SM2, SM3 |  |
| Rafferty (2009) | Journal article | Electronic search | USA | Urban | Public | Elem. | MBL | More than one | MPR | NR | 16-30 | SM2, SM3, SE1, SE2 |  |
| Roberts (2019) | Journal article | Hand search | USA | Rural | Public | High | ABAB | Researcher obs. | ST, MPR, SMS | Researcher obs. | <15 | SA2, SA3, SA4, SM1, SM2, SM3, SE2, SE4 |  |
| Rock (2007) | Journal article | Electronic search | USA | NR | Public | Elem. | ABAB | More than one |  | NR | 16-30 | SM2, SM3, SE2 |  |
| Romans (2017) | Thesis/ Dissertation | Grey literature | USA | Rural | Public | High | ABAB | Researcher obs. | SMS | Checklists | <15 | SA4, SM2, SM3, SE2, SE3 |  |
| Rooney (1998) | Journal article | Electronic search | USA | Urban | Public | Middle | ABAB | Researcher obs. | EX | NR | 31-45 | SM2, SM3, SE2 |  |
| Rosenbloom (2016) | Journal article | Reference list search | USA | Urban | Public | Elem. | ABAB | Researcher obs. | MPR, SMS | Researcher obs. | <15 | SM1, SM2 |  |
| Schaeffer (1989) | Thesis/ Dissertation | Grey literature | USA | Urban | Public | Elem. | MBL | Researcher obs. | MPR | NR | 16-30 | SA4, SM1, SM2, SM3, SE2, SE4 |  |
| Schardt (2019) | Journal article | Hand search | USA | Urban | Charter | Elem. | MBL | More than one | MPR | Researcher obs. | 16-30 | SA4, SM2, SM3, SE2 |  |
| Schmitt (2009) | Thesis/ Dissertation | Electronic search | USA | NR | Public | Elem. | MBL | Researcher obs. | NR | Checklists | <15 | SM2, SM3, SE2 |  |
| Smith (2000) | Journal article | Electronic search | USA | NR | Alt. | Middle | ABAB | Researcher obs. | MPR, SMS | NR | <15 | SM1, SM2, SM3, SE1, SE3 |  |
| Stahr (2006) | Journal article | Reference list search | USA | Urban | Alt. | Elem. | ABAB | Researcher obs. | MPR, SMS | NR | 16-30 | SA3, SA4, SM2, SM3, SE2 |  |
| Szwed (2013) | Journal article | Electronic search | USA | Urban | Public | Elem. | ABAB | Researcher obs. | MPR | Researcher obs. | <15 | SM2, SM3, SE2 |  |
| Vogelgesang (2016) | Journal article | Electronic search | USA | NR | Public | Elem. | ABAB | Researcher obs. | MPR, SMS | Researcher obs. | 31-45 | SM1, SM2, SM3, SE2 |  |
| Vucina (2017) | Thesis/ Dissertation | Grey literature | USA | NR | Other | High | ABAB | Researcher obs. | MPR, SMS | Researcher obs. | <15 | SM2, SM3, SE2 |  |
| Warnke (2003) | Thesis/ Dissertation | Grey literature | USA | Urban | Public | Elem. | ABAB | Researcher obs. | MPR | NR | 46+ | SM2, SM3 |  |
| Wills (2014) | Journal article | Electronic search | Canada | Suburb. | Public | High | ABAB | Researcher obs. | MPR, SMS | Researcher obs. | <15 | SM2, SM3 |  |
| Wolfe (2000) | Journal article | Electronic search | USA | Urban | NR | Elem. | ABAB | More than one | MPR, SMS | More than one | 16-30 | SM2, SM3, SE1 |  |
| Wood (1998) | Journal article | Electronic search | USA | Urban | Charter | Middle | MBL | More than one | MPR, SMS | NR | 16-30 | SM1, SM2, SM3, SE2 |  |
| Wood (2002) | Journal article | Electronic search | USA | NR | Charter | High | MBL | More than one | MPR | More than one | 31-45 | SM2, SM3, SE2 |  |
| Note: NR = Not reported, Alt. = Alternative school, Comm. = Community, Int. = Intervention, Meas. = Measurement, obs. = observations, MBL = multiple baseline  Intervention training abbreviations: ST = Sequenced training, MPR = Modeling, practice, rehearsal, SMS = Focused on SM skills (examples/non-examples), EX = Explicit (lessons, manual)  Intervention component abbreviations: SA1 = self-select target behavior, SA2 = Self-define target behavior, SA3 = Self-determine performance goal[s], SA4 = Self-identify reinforcer, SM1 = Self-prompt reflect on target behavior, SM2 = Self-observe target behavior, SM3 = Self-record the observation, SE1 = Self-chart obs., SE2 = Self-appraise performance, SE3 = Self-administer primary reinforcers, SE4 = Self-administer secondary reinforcers | | | | | | | | | | | | | |

## APPENDIX E: RISK OF BIAS PER EACH INCLUDED SCD STUDY


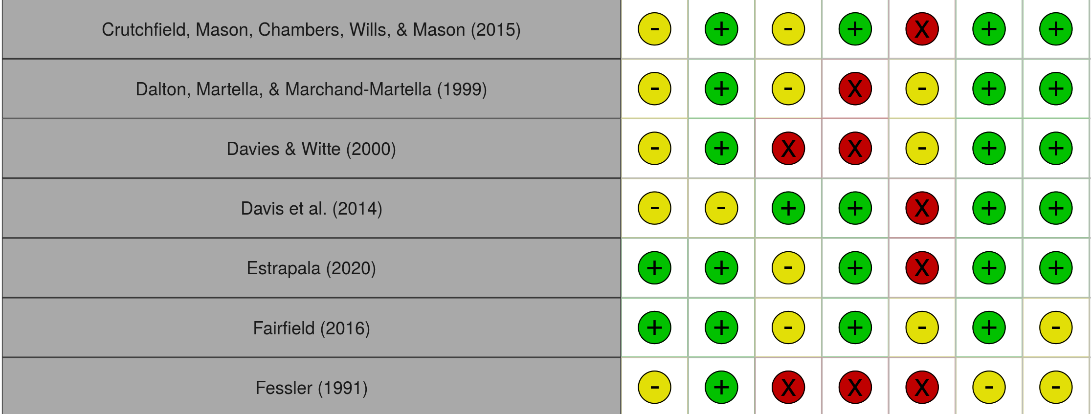

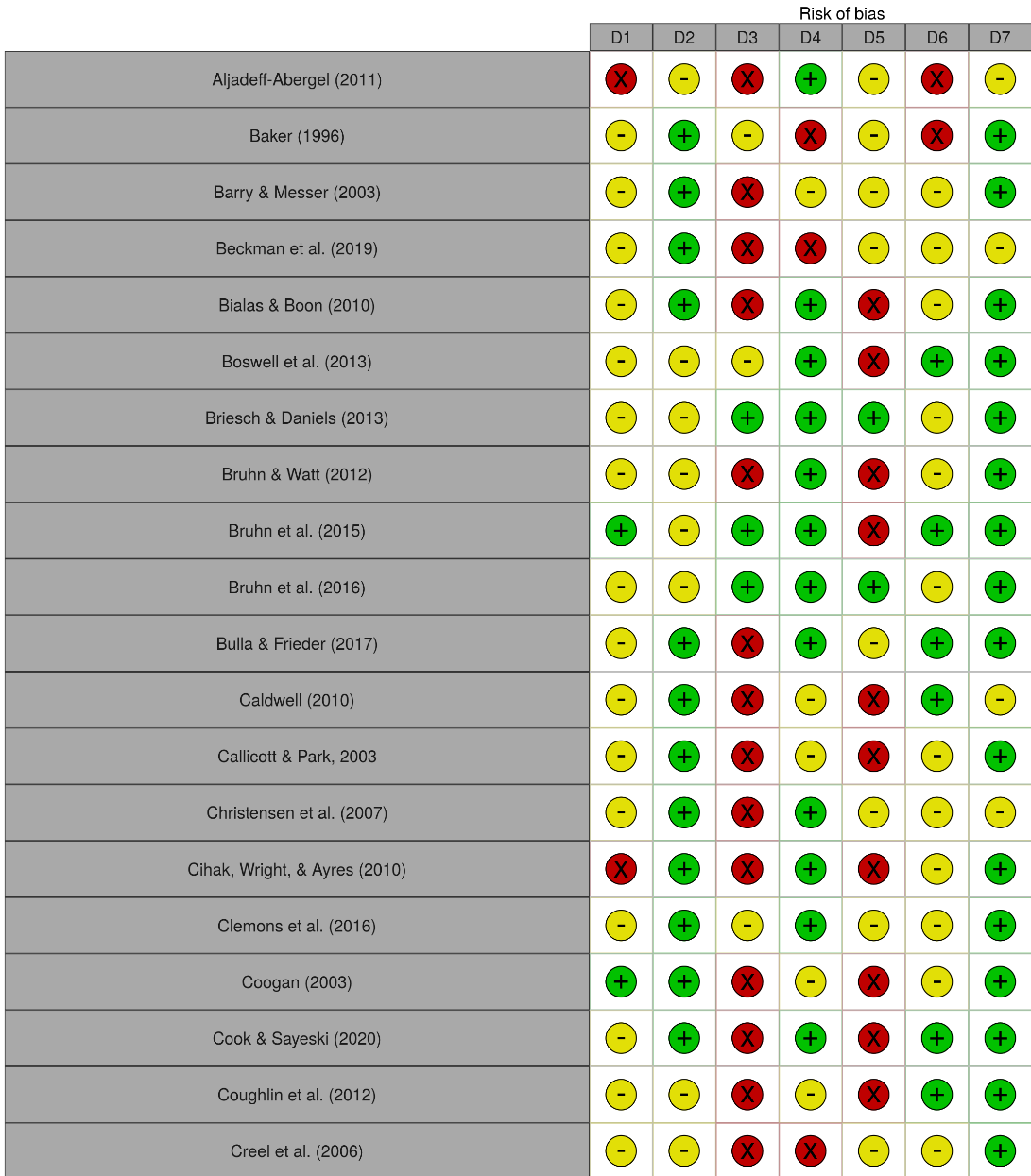


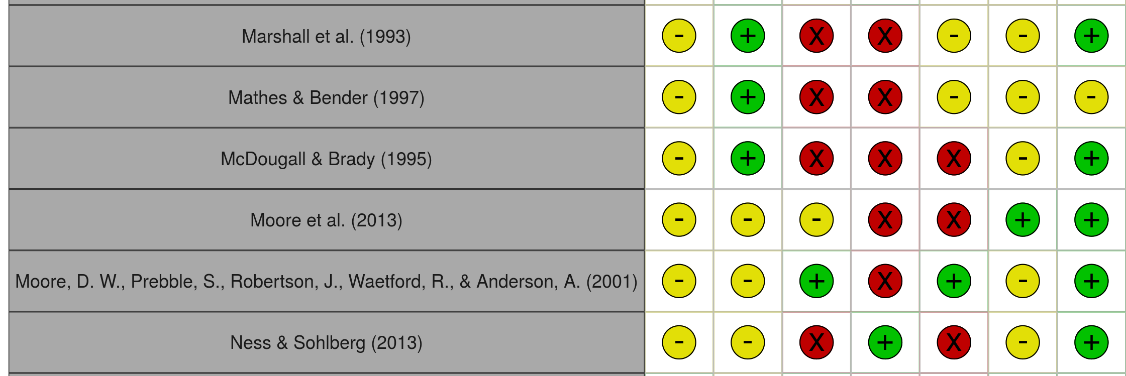

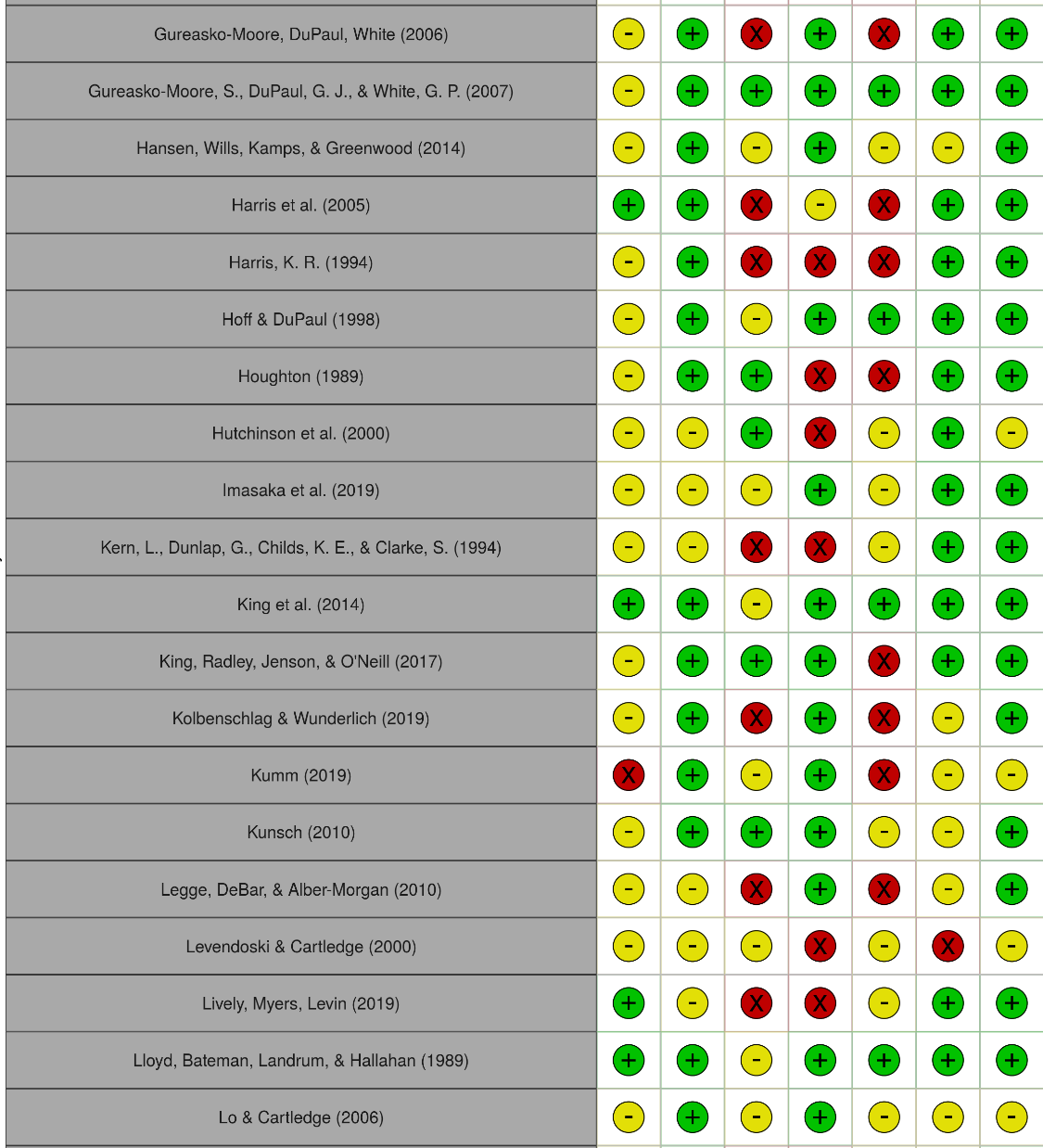


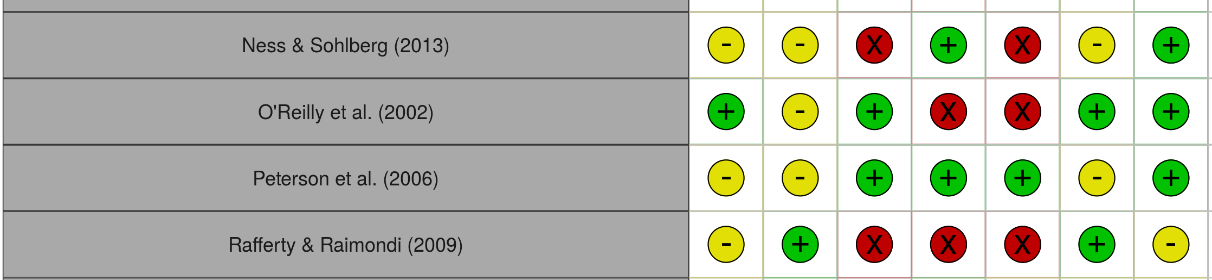


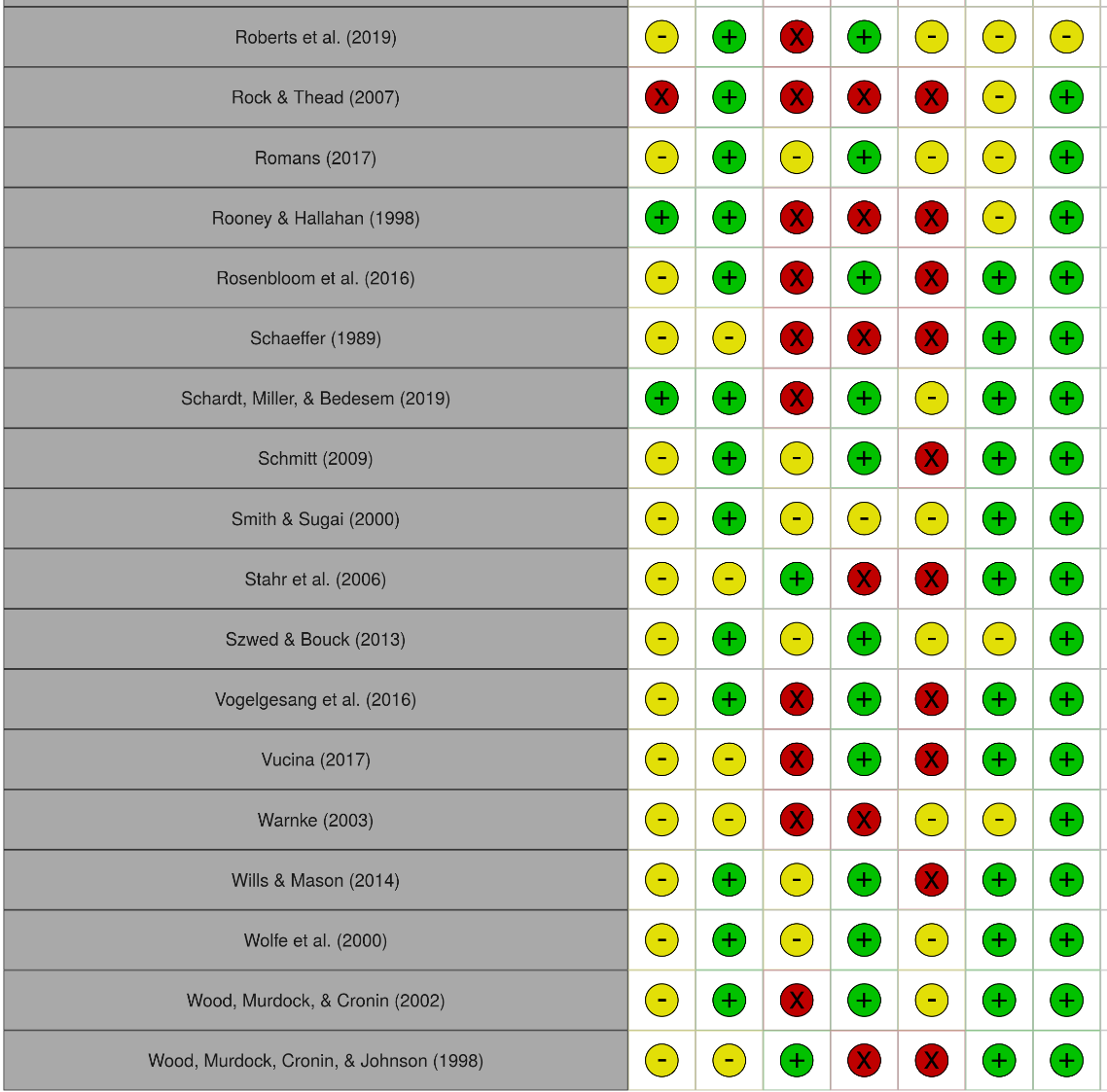

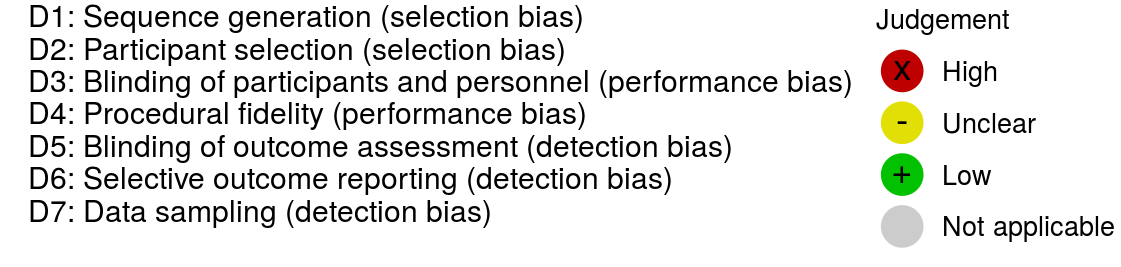


##
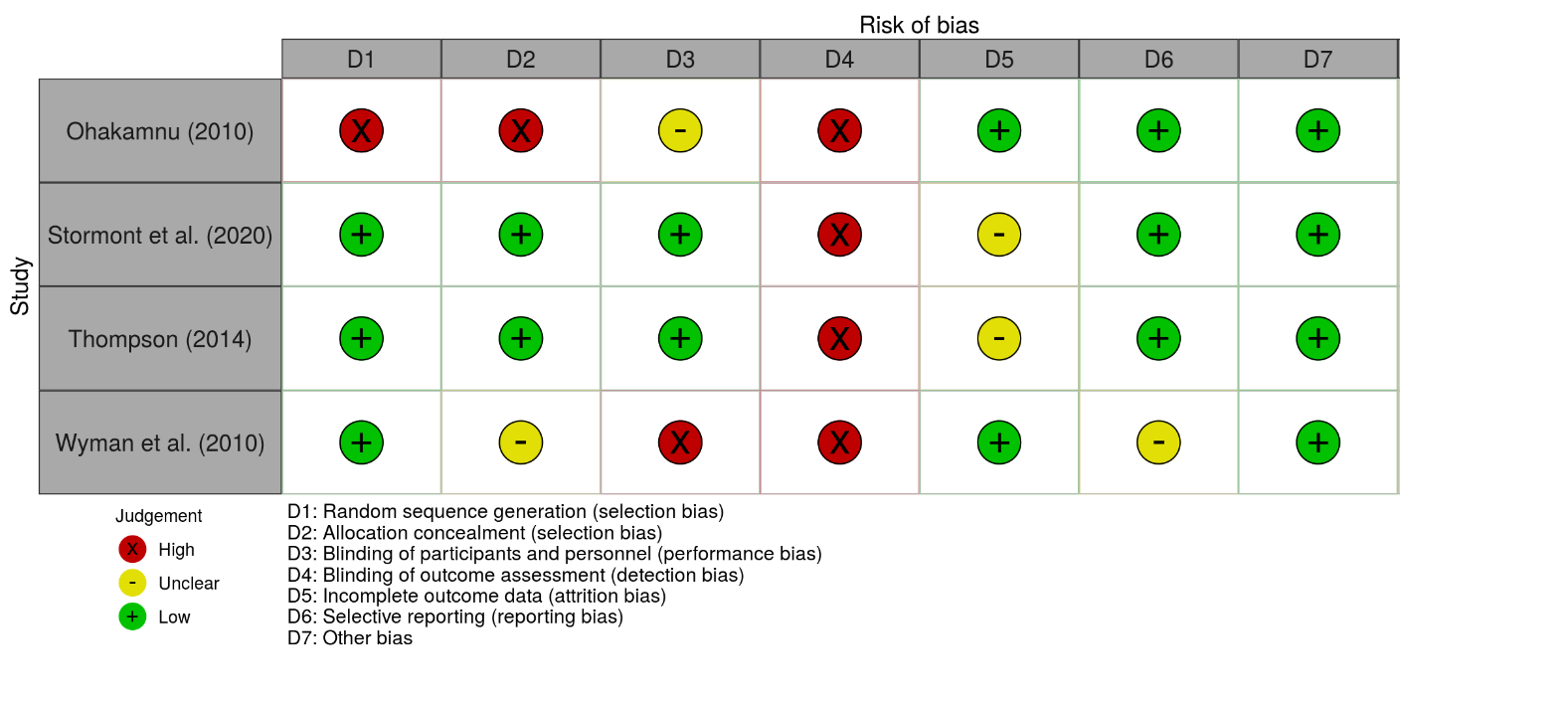
APPENDIX F: RISK OF BIAS FOR INCLUDED GROUP-DESIGN STUDIES
